# Dopamine depletion leads to pathological synchronization of distinct basal ganglia loops in the beta band

**DOI:** 10.1371/journal.pcbi.1010645

**Published:** 2023-04-27

**Authors:** Andrea Ortone, Alberto Arturo Vergani, Mahboubeh Ahmadipour, Riccardo Mannella, Alberto Mazzoni

**Affiliations:** 1 Dipartimento di Fisica, Università di Pisa, Pisa, Italy; 2 The BioRobotics Institute, Scuola Superiore Sant’Anna, Pontedera, Italy; 3 Department of Excellence in Robotics and AI, Scuola Superiore Sant’Anna, Pisa, Italy; Brandeis University, UNITED STATES

## Abstract

Motor symptoms of Parkinson’s Disease (PD) are associated with dopamine deficits and pathological oscillation of basal ganglia (BG) neurons in the *β* range ([12-30] Hz). However, how dopamine depletion affects the oscillation dynamics of BG nuclei is still unclear. With a spiking neurons model, we here capture the features of BG nuclei interactions leading to oscillations in dopamine-depleted condition. We highlight that both the loop between subthalamic nucleus (STN) and Globus Pallidus pars externa (GPe) and the loop between striatal fast spiking and medium spiny neurons and GPe display resonances in the *β* range, and synchronize to a common *β* frequency through interaction. Crucially, the synchronization depends on dopamine depletion: the two loops are largely independent for high levels of dopamine, but progressively synchronize as dopamine is depleted due to the increased strength of the striatal loop. The model is validated against recent experimental reports on the role of cortical inputs, STN and GPe activity in the generation of *β* oscillations. Our results highlight the role of the interplay between the GPe-STN and the GPe-striatum loop in generating sustained *β* oscillations in PD subjects, and explain how this interplay depends on the level of dopamine. This paves the way to the design of therapies specifically addressing the onset of pathological *β* oscillations.

## Author summary

PD is associated with the death of neurons generating a particular neurotransmitter: the dopamine. Motor symptoms of PD, on the other hand, are known to be due to dysfunctions in a particular subcortical area of the brain: the BG network. In particular, the BG network develops pathological oscillations in the *β* ([12–30] Hz) frequency range. What is unclear is the mechanism through which dopamine depletion leads to these oscillations. In this work we developed a BG network model and we found that the actual reason for these abnormal oscillations is the synchronization of two loops within the network that are individually oscillating in the *β* range. For high levels of dopamine the two loops are decoupled and the oscillation power is low. Conversely. when dopamine is depleted (as in PD) the two loops synchronize and originate the pathological oscillations associated with motor symptoms.

## Introduction

**Fig 1:**
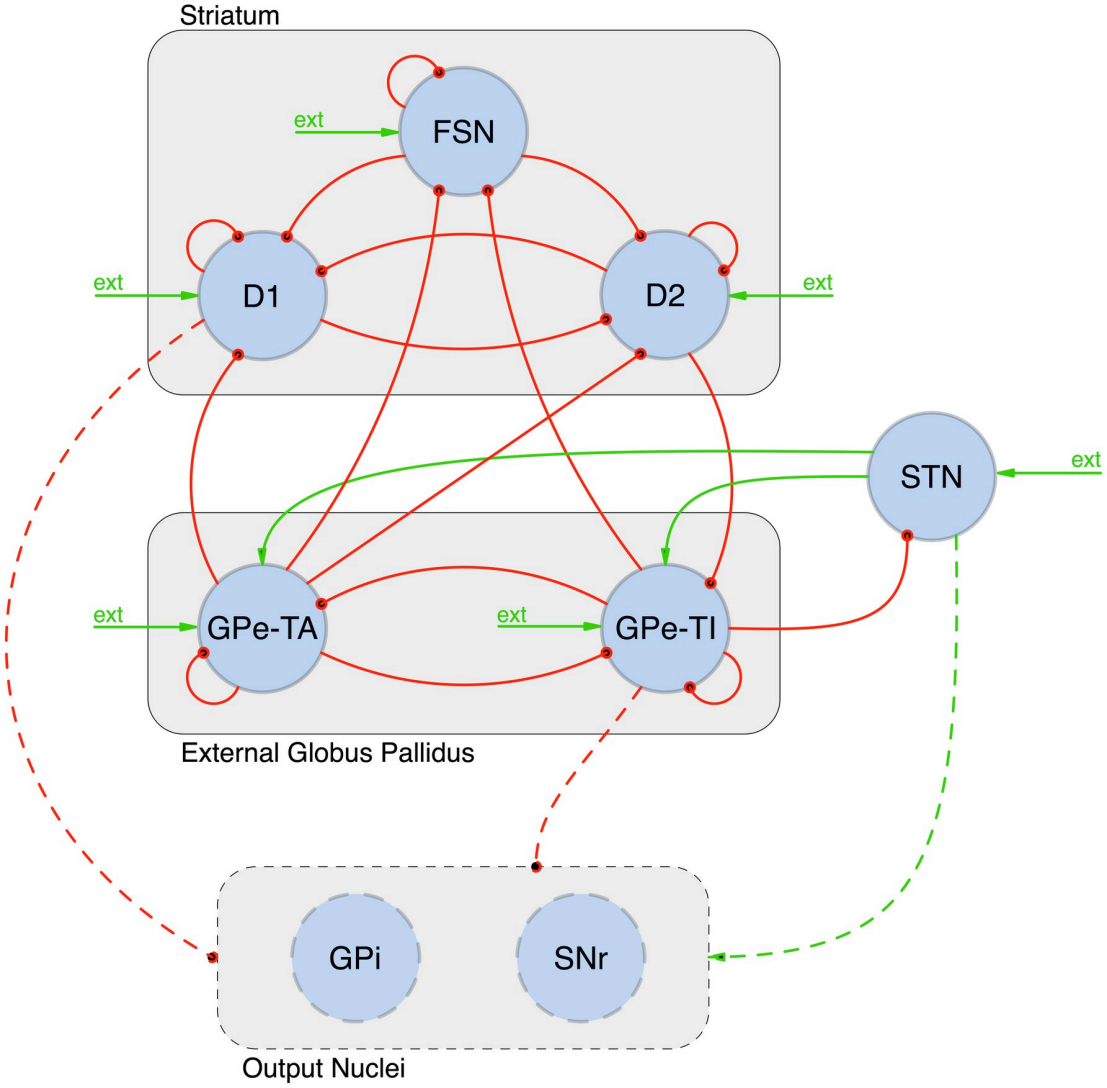
Architecture of the adopted Complete Model of the BG. FSN, D1 and D2: striatal Fast Spike Neurons, and medium spiny neurons with D1 and D2 dopamine receptors; GPe-TA and GPe-TI: globus pallidus externa type A and type I; STN: subthalamic nucleus; GPi: globus pallidus interna; SNr: substantia nigra pars reticulata; ext: external poissonian input. By convention red/green arrows are inhibitory/excitatory projections. Dashed elements have not been included in our model.

The basic architecture of the BG network (see Fig 1) consists of the striatum (STR), the globus pallidus, divided into pars interna (GPi) and pars esterna (GPe), the substantia nigra (reticulata SNr and compacta SNc) and the STN [1, 2]. In the STR there are several interacting cell types such as dopamine-excited D1 neurons, dopamine-inhibited D2 neurons and Fast Spiking Neurons (FSN).

PD is a common neurodegenerative disease affecting about 0.3% of the world’s population [3]. Etiologically, PD follows the progressive death of dopaminergic neurons in the SNc. The consequent condition of dopamine depletion leads to an alteration of the balance between D1 excitation and D2 inhibition [4] and this reverberates over the whole network. The main motor symptoms of PD (akinesia [5], bradykinesia [6], tremor [7], freezing of gait [8, 9]) correlate with this dopamine deficiency. Following the condition of dopamine depletion and the consequent alteration of the striatal activity (higher spiking rate in D2 and lower in D1 [10, 11]), pathological *β* oscillations [12 − 30] Hz emerge in the STR [12–15], in GPi and GPe [16, 17], and in the STN [18, 19]. Experimental recordings [20–22] highlighted that such pathological activity is not characterized by constant intensity, but consists of phasic bursts. The presence of *β* oscillations in correspondence with dopamine deficiency is common in the human species, but has been observed also in monkeys [23] and rats [24, 25]. Of note, in mice, dopamine depletion is associated instead with *δ* (0.5–4 Hz) oscillations [26].

Despite the wealth of studies in the subject, the origin of these *β* activities is still debated.

Some hypotheses argue that the oscillations depend on the interaction between GPe and STN [27]. In support of this hypothesis, it is known that the architecture of the STN-GPe circuit is prone to generate oscillatory behaviors [28]: STN is a glutamatergic nucleus projecting substantially to GPe and GPe is a gabaergic nucleus projecting feedback to STN [29]. Moreover, in PD, these nuclei present prominent and coherent oscillations in the firing rates [24, 28] and are effective targets of Deep Brain Stimulation (DBS) therapy [30–32]. However, whether *β* band activity can be produced in the STN–GPe circuit is still debated [33]. Several computational models investigated the STN-GPe *β* band loop: first, Gillies at al [34] highlighted that *β* oscillations could emerge from the interaction between GPe and STN through neural mass models; later, Kumar et al [35] showed that pathological *β* activity could emerge from the same loop as a consequence of increased inhibitory input from the D2 population through a spiking neurons model. Further explorations of this hypothesis can also be found in [36–39].

Alternative explanations for the experimental observations are that the cortex [40, 41] or the striatum [42] might be a source of *β* oscillations. In accordance with the former hypothesis, Van Albada et al. [43] suggested a cortico-thalamic loop source of *β* oscillations, which spread into the BG. Conversely, the latter hypotheses is supported by evidences of altered striatal interaction associated with *β* oscillations following dopamine depletion [44].

The fourth hypothesis is the one of a prominent role of the interaction between GPe and STR: experimental recordings in rats [45–49] and mice [50–53] suggest that the GPe nucleus and the pallido-striatal pathway could play a major role. In vivo observations of pauses in FSN support this hypothesis as transient drop in the activity might arise from interactions with GPe [54] (see also [55, 56] for an extended review). From a computational point of view, a model of the GPe-striatal circuit has been developed by Corbit et al [57], who investigated the onset of pathological *β* oscillations in a subnetwork consisting of the closed loop set up by the inhibitory synapses FSN → D2-MSN → GPe → FSN.

A recent work by Mallet and colleagues [49] provides, through optogenetic perturbations, a convenient summary of the role of cortical inputs, GPe and STN activity in modulating abnormal *β* oscillations in PD. The authors show that opto-inhibition of cortical inputs or STN did not suppress such oscillations, that were instead suppressed by GPe inhibition: to date, no computational model is able to comprehensively explain the observed dynamics.

To reach this goal, we propose a comprehensive model including all the loops, starting from the network developed by Lindahl and Kotaleski [58] which includes all major nuclei and connections of the BG. In this context, we identify the major sources of *β* activity, investigate the role of their interaction and highlight the effects of dopamine depletion.

## Materials and methods

### 1.1 Basal ganglia network model

Our model of the BG (Fig 1) includes:

- three striatal populations: D1-type dopamine receptor Medium Spiny Neurons (D1-MSN); D2-type dopamine receptor Medium Spiny Neurons (D2-MSN) and a population of Fast Spiking (inter-)Neurons (FSN);
- the Globus Pallidus external part (GPe) divided into two populations that will be labelled as GPe-TA (characterized by a lower discharge rate and by a negligible input from striatal populations) and GPe-TI (with a higher activity and receiving input from D2);
- the subthalamic nucleus (STN).

Each population presents specific size (see Table 1) and neuron model (see Section 1.2). Population sizes were adapted from the work of Lindahl and Kotaleski [58], based on rodents data [46, 59–61]: the ranking of populations among nuclei is the same, but we rescaled all of them for computational time reasons. Single neurons within connected populations are randomly associated according to specific connection probabilities (see Section 1.2). Each neuron receives inputs from within the network and from other brain regions (mainly the cortex) which have not been explicitly included in our model. These external inputs have been modeled as independent poissonian trains of pulses. The mean rates *ν*_*ext*_ of these signals have been adjusted for each population in order to ensure realistic population firing rates: FSN [1020] Hz ([55, 62]), D1 and D2 (MSN) [0.5–2.5] Hz ([63]), GPe-TI [40–60] Hz ([45]), GPe-TA [5–15] Hz ([45]) and STN [12–20] Hz ([24]).

**Table 1:** Reference size and neuron model of each population. Note that sizes have been modulated in a subset of the analysis through the *n* parameter.

SubNetwork	*N*	model
D1 (MSN)	6000	aqif_cond_exp
D2 (MSN)	6000	aqif_cond_exp
FSN	420	aqif2_cond_exp
GPe-TA	264	aeif_cond_exp
GPe-TI	780	aeif_cond_exp
STN	408	aeif_cond_exp

Note that the GPi and the SNr nuclei have not been included in the simulated model since they do not present direct feedback to the other populations in the BG and hence cannot contribute to the generation of oscillations.

### 1.2 Neuron models

All neurons in the adopted network are adaptive, conductance based, point neurons [64–67]. The STN, GPe-TI and GPe-TA populations are modelled as adaptive exponential neurons (aeif_cond_exp model in the code implementation) [68] and the dynamics of their membrane potential is governed by:

$$\begin{array}{c} C_{m}\frac{{\text{d}}^{}V}{\text{d}t^{}}=-g_{L}\left(V-E_{L}\right)-g_{ex}\left(V-E_{ex}\right)-g_{in}\left(V-E_{in}\right)+g_{L}\Delta T\;\text{exp}\;(\frac{V-V_{th}}{\Delta T})-w+I_{e} \end{array}$$

The striatal populations are modelled as adaptive quadratic neurons (aqif_cond_exp or aqif2_cond_exp model) [69] and their membrane potential evolves according to:

$$\begin{array}{c} C_{m}\frac{{\text{d}}^{}V}{\text{d}t^{}}=-g_{ex}\left(V-E_{ex}\right)-g_{in}\left(V-E_{in}\right)+k\left(V-E_{L}\right)\left(V-V_{th}\right)-w+I_{e} \end{array}$$

In both cases, the membrane potential is reset to *V*_*reset*_ after each postsynaptic spike and excitatory and inhibitory conductances present exponential decays:

$$\{\begin{array}{l} \tau_{ex}\frac{\text{d}g_{ex}}{\text{d}t}=-g_{ex}+\tau_{ex}\sum_{\text{ex}.\;\text{input}\;t_{i}}g_{i}\delta (t-t_{i}-t_{r})\\ \tau_{in}\frac{\text{d}g_{in}}{\text{d}t}=-g_{in}+\tau_{in}\sum_{\text{in}.\;\text{input}\;t_{i}}g_{i}\delta (t-t_{i}-t_{r}) \end{array}$$

The evolution of the variable *w*, accounting for neural adaptation phenomena [70, 71] is governed by:

$$\begin{array}{c} \tau_{w}\frac{{\text{d}}^{}w}{\text{d}t^{}}=-w+a\left(V-E_{L}\right)+b\;\tau_{w}\sum_{\text{spikes}\;t_{i}}\delta \left(t-t_{i}\right) \end{array}$$

in the aeif_cond_exp and aqif_cond_exp model, while by:

$$\begin{array}{c} \tau_{w}\frac{\text{d}w}{\text{d}t}=b\;\tau_{w}\sum_{\text{spikes}\;t_{i}}\delta \left(t-t_{i}\right)+ \end{array}\left\{\begin{array}{ll} -w+a{\left(V-V_{b}\right)}^{3} & \text{if}\;V<V_{b}\\ -w & \text{otherwise} \end{array}\right.$$

in the case of the aqif2_cond_exp model.

To introduce variations in each neural population, the synaptic weight of the external poissonian input is not equal in all neurons. Rather, it is assumed to be uniformly distributed around its central value with an amplitude equal to the dev-ext-weight parameter. The adopted values of the parameters of each neuronal population are summarized in Table 2. The connectivity properties (delays, connection probabilities and synaptic weights) are reported in Table 3. These values have been adapted from the work of Lindahl and Kotaleski [45, 58, 72], here simplified by neglecting synaptic plasticity, spatial restrictions on the connected populations and dopamine effects different from the ones described in Section 1.5.

**Table 2:** Adopted values of the parameters of each neuronal population. (*): for the FSN population the units of *a* is $\frac{\text{nS}}{{\text{mV}}^{2}}$.

Parameter	Unit	D1	D2	FSN	GPTI	GPTA	STN
*C*_*m*_	pF	15.2	15.2	80.0	40.0	60.0	60.0
*E*_*L*_	mV	-78.2	-80.0	-80.0	-55.1	-55.1	-80.2
*E*_*ex*_	mV	0.0	0.0	0.0	0.0	0.0	0.0
*E*_*in*_	mV	-74	-74	-74	-65	-65	-84
*τ*_*ex*_	ms	12.0	12.0	12.0	10.0	10.0	4.0
*τ*_*in*_	ms	10.0	10.0	10.0	5.5	5.5	8.0
*V*_*th*_	mV	-29.7	-29.7	-50.0	-54.7	-54.7	-64.0
*I*_*e*_	pA	0.0	0.0	0.0	12.0	1.0	5.0
*t*_*ref*_	ms	0.0	0.0	0.0	0.0	0.0	0.0
*V*_*reset*_	mV	-60	-60	-60	-60	-60	-70
*a*	nS	-20	-20	0.025*	2.5	2.5	0.0
*b*	pA	67.0	91.0	0.0	70.0	105.0	0.05
*τ*_*w*_	ms	100.0	100.0	5.0	20.0	20.0	333.0
*V*_*peak*_	mV	40.0	40.0	25.0	15.0	15.0	15.0
Δ*T*	mV				1.7	2.55	16.2
*g*_*L*_	nS				1.0	1.0	10.0
*k*	$\frac{\text{nS}}{\text{mV}}$	1.0	1.0	1.0			
*V*_*b*_	mV			-55			
*ν*_*ext*_	kHz	1.12	1.083	0.944	1.53	0.17	0.5
dev-ext-weight	nS	0.05	0.05	0.05	0.05	0.05	0.05

**Table 3:** Connectivity properties of the adopted model of the BG. Type I represents inhibitory connections while type E represents excitatory connections.

Source	Target	Probability	Delay [ms]	Type	synaptic weight [nS]
D1	D1	0.0607	1.7	I	0.12
	D2	0.0140	1.7	I	0.30
D2	D1	0.0653	1.7	I	0.36
	D2	0.0840	1.7	I	0.20
	GPTI	0.0833	7.	I	1.28
FSN	D1	0.0381	1.7	I	6.60
	FSN	0.0238	1.0	I	0.50
	D2	0.0262	1.7	I	4.80
GPTI	GPTI	0.0321	1.0	I	1.20
	GPTA	0.0321	1.0	I	0.35
	FSN	0.0128	7.0	I	1.60
	STN	0.0385	1.0	I	0.08
GPTA	D1	0.0379	7.0	I	0.35
	D2	0.0379	7.0	I	0.61
	FSN	0.0379	7.0	I	1.85
	GPTA	0.0189	1.0	I	0.35
	GPTI	0.0189	1.0	I	1.20
STN	GPTA	0.0735	2.0	E	0.13
	GPTI	0.0735	2.0	E	0.42
ext	D1	1.0	0.0	E	0.45
	D2	1.0	0.0	E	0.45
	FSN	1.0	0.0	E	0.50
	GPTI	1.0	0.0	E	0.25
	GPTA	1.0	0.0	E	0.15
	STN	1.0	0.0	E	0.25

Hereinafter, we will refer to the described network of the BG as the Complete Model. Note that the results of the model are not critically dependent on the exact values of the network parameters (see Section 6 in S1 Text).

### 1.3 Selection of relevant *β* oscillators

In order to identify the more relevant structures generating *β* oscillations, the following strategy has been pursued. Starting from the Complete Model (Fig 1), the relevance of each connection in generating oscillations in the *β* regime has been quantified referring to the decrease in the mean intensity of *β* oscillations following its elimination. In particular, for each connection *Source* → *Target* (*S* → *T*) in the adopted model, a simulation has been performed with the following properties:

- an *auxiliary* subnetwork S* has been introduced with the same neuron model and size of *S* but with no input sources other than the external one;
- the parameter *I*_*e*_ of the S* subnetwork has been adjusted so that the mean spiking-rate of S* neurons was close to the one of the *S* population;
- the probability of connection between *S* and *T* neurons is set to 0;
- the *S* → *T* connections are replaced by S* → *T* connections with the same probability and synaptic weight.

The relevance of the *S* → *T* connection has been thereby quantified by means of the ratio *R*(*S* → *T*) between the mean PSD (see below Section 1.7 for details) with or without the (*S* → *T*) connection:

$$\begin{array}{c} R\left(S\to T\right)=\frac{\sum_{p}\;\text{Mean}\;\beta \;\text{PSD}\;\text{of}\;p\;\text{in}\;\text{the}\;\text{simulation}\;\text{with}\;\text{the}\;S^{*}\to T\;\text{conn.}}{\sum_{p}\;\text{Mean}\;\beta \;\text{PSD}\;\text{of}\;p\;\text{in}\;\text{the}\;\text{simulation}\;\text{with}\;\text{the}\;S\to T\;\text{conn.}} \end{array}$$ (1)

where *p* indicates the different neuronal populations. The connections whose ratio was lower were considered as more relevant in the generation of *β* oscillations. The major oscillators in the *β* regime have been identified on the basis of the selected connections. Self-inhibition connections have not been considered in this analysis since they generate oscillations at much higher frequencies [73].

### 1.4 Simplified Model of the Basal Ganglia and role of the coupling parameter *ε*

As from our analysis emerged that two oscillators are mainly responsible for the onset of the pathological activity, we developed an *ad hoc* network to analyze the properties and the consequences of their interaction (see Section 2.3 and figures therein).

The GPe-TI nucleus is present in both oscillators, hence its neurons have been equally divided in two populations (GPTI-A and GPTI-B) belonging to the two different loops: neurons in GPTI-A are reciprocally connected to STN, while those in GPTI-B receive inputs from D2 and send outputs to FSN. These connections present connection probabilities that are equal to the ones in the Complete Model (see Table 3).

In contrast, in order to modulate the coupling between the two oscillators, *inter*-loop connections present connection probabilities which are modulated by a coupling parameter *ε*. Particularly, for each pair (*S*,*T*), the connection probability of each *S* neuron with each *T* neuron is set equal to:

$$\begin{array}{c} p\left(\varepsilon ,S\to T\right)=\varepsilon p_{1,S\to T} \end{array}$$ (2)

where *p*_1,*S*→*T*_ is the probability characterizing the corresponding connection in the Complete Model (Table 3).

For every connection, synaptic weights are the same as in the Complete Model (and are not affected by *ε*). As a result, for *ε* = 0 the two loops are completely independent, while for *ε* = 1 the network presents realistic connectivity properties. Further, in order to ensure that the overall inputs to each nucleus remain the same when *ε* is changed, the *auxiliary* populations GPTI*, D2* and STN* have been introduced. Neurons in these nuclei receive constant and independent external input such that their mean spiking rates are equal to the mean spiking rates of the corresponding non-*auxiliary* populations. While the synaptic weights of the *auxiliary* connections are the same as the corresponding real ones in the original network, the connection probabilities S* → *T* are set equal to:

$$\begin{array}{c} p\left(\varepsilon \right)=(1-\varepsilon )p_{1,S\to T} \end{array}$$

Hence, for *ε* = 1 the auxiliary nuclei do not play any role; otherwise, they become external drives meant to compensate for the input that is not received from the *real* presynaptic populations. The neuron models and the parameters of the *auxiliary* populations are the same of the corresponding *real* populations (Table 2), except for *ν*_*ext*_ and the size *N*, which is halved. As a consequence, the connection probabilities D2*→GPTI-A and STN*→GPTI-B are doubled.

Since most nuclei in this simplified network receive less inhibition in comparison to the Complete Model, some adaptations in the external current *I*_*e*_ and the external poissonian input *ν*_*ext*_ have been introduced in order to keep the mean activity of the populations in the correct regime. Further, since all the non-*β* oscillators have been eliminated, *β* activity with the original connection weights is dominant for all *ε*; to study the process of synchronization in these conditions, it has been necessary to modulate the intensities of the connections constituting the two loops. The complete list of the introduced adaptations are listed in Table A in S1 Text.

In the following, and in contrast to the original Complete Model (see Section 1.1), the network obtained after the listed assumptions and simplifications will be labelled Simplified Model.

### 1.5 Modeling of the condition of Dopamine depletion

**Fig 2:**
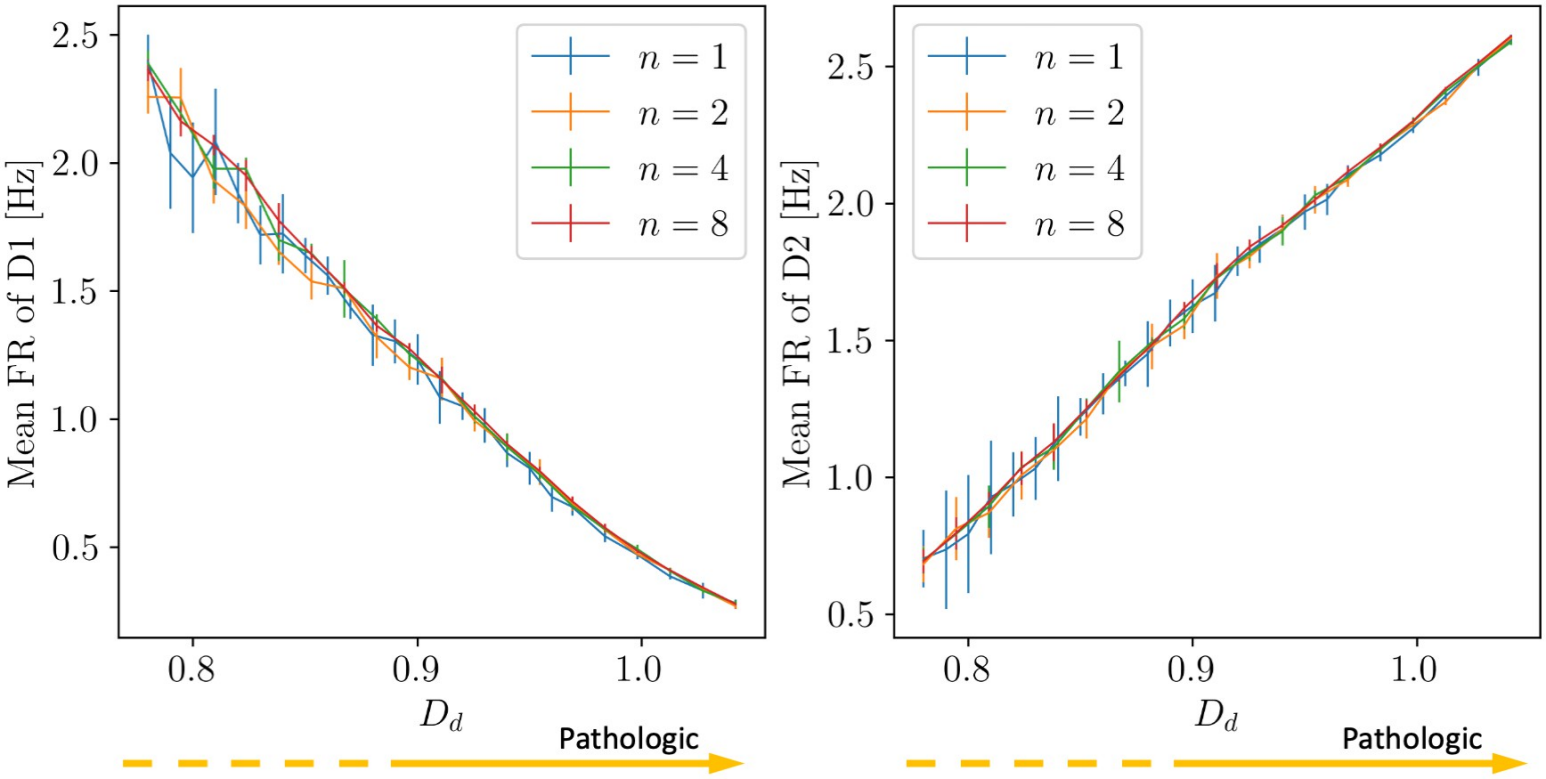
D1 and D2 firing rates as a function of dopamine depletion. Alterations in the mean firing rates of D1 and D2 neurons as a result of the adopted modelization of dopamine depletion for different values of population size *n* (see Section 1.8).

We aimed at modeling the effects of dopamine depletion on the firing rate of D1 and D2 populations [10, 11]. To achieve an increase of the D2 population and a complementary decrease of the D1 population it was sufficient to modulate the intensity of the external input rate towards the D2 population (and the corresponding auxiliary population D2*) according to:

$$\begin{array}{c} \nu_{\text{ext}}\left(D_{d}\right)=D_{d}\;\nu_{\text{ext},1} \end{array}$$ (3)

where *ν*_ext,1_ is the reference value of the input rate (see Table 2) and *D*_*d*_ is the parameter regulating the severity of the condition of dopamine depletion (the higher is *D*_*d*_, the more severe is the condition). Throughout the analysis, *D*_*d*_ varies in an interval such that the activities of D1 and D2 range in the [∼ 0.5 Hz− ∼ 2.5 Hz] interval (see Fig 2). The effects of dopamine depletion will be studied in both the Complete and Simplified Model.

With this assumption, we neglect the effects of dopamine depletion on the connectivity properties of the network [57, 74–76]: the consequences of including these latter alterations are addressed in Section 5 in S1 Text.

### 1.6 Modeling of optogenetic-driven activity modulations

To reproduce the conditions described in [49] we needed to introduce in our model an analogous of the activity modulations induced by the experimenters through optogenetic stimulation in a Parkinsonian mouse model. In particular, the authors focused on *i*) opto-inhibition of motor cortex input, *ii*) both opto-inhibition and opto-excitation of STN activity, *iii*) opto-inhibition of GPe activity. Coherently, we analyzed the behavior of our model in pathological conditions (*D*_*d*_ = 1.03) in response to the following activity perturbations.

First, we modeled the motor cortex opto-inhibition (point *i*) as a reduction in the mean of the poissonian external input to the STN and a portion of the striatal populations. Since the dendritic domain of individual subthalamic neurons covers large parts of the whole nucleus [77], we modulated the external input of the whole STN according to:

$$\begin{array}{c} \nu_{ext,\text{STN}}^{\prime }=0.75\cdot \nu_{ext,\text{STN}} \end{array}$$

Since the striatum receives inputs from all major cortical areas and these inputs are topographically arranged [78, 79], we partitioned each striatal population into a 20% of the neurons mainly innervated by the motor cortex (D1_*m*_, D2_*m*_, FSN_*m*_) and the remaining 80% of the neurons mainly innervated by other cortical areas (D1_*nm*_, D2_*nm*_, FSN_*nm*_). Hence, we modulated their external input rate according to:

$$\begin{array}{cccc} \nu_{ext,\text{D1}{}_{m}}^{\prime } & =0.25\cdot \nu_{ext,\text{D1}}\;\; & \nu_{ext,\text{D1}{}_{nm}}^{\prime } & =\nu_{ext,\text{D1}}\\ \nu_{ext,\text{D2}{}_{m}}^{\prime } & =0.25\cdot \nu_{ext,\text{D2}} & \nu_{ext,\text{D2}{}_{nm}}^{\prime } & =\nu_{ext,\text{D2}}\\ \nu_{ext,\text{FSN}{}_{m}}^{\prime } & =0.25\cdot \nu_{ext,\text{FSN}} & \nu_{ext,\text{FSN}{}_{nm}}^{\prime } & =\nu_{ext,\text{FSN}} \end{array}$$

Then, we modeled the STN opto-inhibition and opto-excitation (point *ii*) modulating the mean of the poissonian external input according to:

$$\begin{array}{c} \nu_{ext,\text{STN}}^{\prime }=\kappa \cdot \nu_{ext,\text{STN}} \end{array}$$

with *κ* ∈ [0.65, 1.7] so that the activity of STN varies in the range [∼ 8, ∼30] Hz, coherently with the conditions considered in [49].

Finally, we modeled the GPe opto-inhibition (point *iii*) modulating the external current *I*_*e*_ to both GPe-TA and GPe-TI populations according to:

$$\begin{array}{cc} I_{e,\text{GPE-TA}}^{\prime } & =I_{e,\text{GPE-TA}}-480\;\text{pA}\\ I_{e,\text{GPE-TI}}^{\prime } & =I_{e,\text{GPE-TI}}-120\;\text{pA} \end{array}$$

so that the activities of the two pallidal populations were coherent with those analysed in [49].

### 1.7 Spectral analysis

We measured the activity of each population as the time series of its firing rate computed over time bins of one millisecond. For each nucleus we performed a constant detrend and computed the Power Spectral Density (PSD) of the activity. In order to reduce the effects of noise, the Welch method has been applied (we employed the signal.spectrogram function from scipy with N_parseg = 2000, N_overlap = 1000 and Tukey window: alpha = 0.25); further, for each case studied, 4 simulations have been performed in order to estimate the standard deviation of each computed quantity.

Starting from the PSD of the activity of each population *i* within the network, we defined:

- its mean frequency of oscillation:
  $$\begin{array}{c} \text{Mean}\;f_{i}=\frac{1}{\int_{m}^{M}P_{i}\left(f\right)\;{\text{d}}^{}f\;}\int_{m}^{M}f\;P_{i}\left(f\right)\;{\text{d}}^{}f\; \end{array}$$ (4)
- its mean value in the *β* regime:
  $$\begin{array}{c} \text{Mean}\;{\text{PSD}}_{i}=\frac{1}{M-m}\int_{m}^{M}P_{i}\left(f\right)\;{\text{d}}^{}f\; \end{array}$$ (5)

where *P*_*i*_(*f*) is the PSD of the nucleus activity. The values of *m* and *M* have been fixed to 8 and 24 Hz respectively, within the *β* regime and coherently with the natural frequencies of the two oscillators.

The Mean PSD_*i*_ is a measure of the intensity of *β* oscillations within each considered nuclei. We highlight that this measure is *biased*: in the presence of constant activity (no *β* oscillations), fluctuations around the mean are present and the related spectrum is affected by noise. In order to eliminate this bias we considered the corrected quantity:

$$\begin{array}{c} {\text{PSD}}_{\;i}^{†}=\text{Mean}\;{\text{PSD}}_{i}-Q\left(\nu_{0,i},N_{i}\right) \end{array}$$

where *Q*(*ν*_0,*i*_, *N*_*i*_) is the Mean PSD of the activity of a population of *N*_*i*_ neurons with constant mean activity *ν*(*t*) = *ν*_0,*i*_. The value of *Q*(*ν*_0,*i*_, *N*_*i*_) has been estimated computing the PSD of a fictitious activity in which the number ${\left\{n_{j}^{i}\right\}}_{j}$ of spiking neurons in each bin is given by independent extractions from a binomial distribution:

p(nji;ν0,i,Ni)=B(nji;ν0,i,Ni)=(Ninji)ν0,inji(1-ν0,i)Ni-nji

Throughout this paper, we highlight the spectral properties of the system of the STN and STR oscillators (the two loops mainly responsible for *β* activity) by the analysis of the spectral properties of the STN and D2 populations respectively. The choice of these nuclei is due to the fact that they set up one (and only one) of the two loops, hence determining a benefit in the interpretability of the results.

### 1.8 Analysis of the limit of large populations

In order to verify whether the properties that we obtained persist in the limit of large populations (the realistic size of each population is approximately 10^3^ times the adopted sizes [59, 80]) we repeated all the analysis with each population size multiplied by a factor *n* ∈ [1, 2, 4, 8]:

$$\begin{array}{c} N_{p}\left(n\right)=n\;N_{p} \end{array}$$

where *p* indicates the different neural populations and *N*_*p*_ is the size of population *p* listed in Table 1. In order to have comparable results for different values of *n*, the PSDs have been computed over *N*_*p*_(1) neurons also in the case of *n* ≠ 1. Further, in case of *n* ≠ 1, all connection probabilities were scaled in such a way that the mean number of presynaptic neurons per target neuron was constant for each connection.

### 1.9 Numerical methods

The code for the simulations has been developed in C++. In combination with this code, a python3 module has been implemented in order to import and preliminarily analyse the results of the simulations. The implemented code is available on GitLab (https://gitlab.com/andrea.ortone/basal_ganglia_model) and the documentation is published on readthedocs.org (https://basal-ganglia-model.readthedocs.io/en/latest). The numerical integration of the equations describing the evolution of each neuron in the simulated network are performed with a fixed time step *h* = 0.1 ms and applying the 4^*th*^ order Runge-Kutta procedure [81]. In order to generate random numbers the PCG library [82] has been employed. The duration of each simulation has been fixed to 10 000 ms plus a warm-up interval of 500 ms which has been eliminated from the analysis.

## Results

With the aim of untangling the dynamics leading to the onset of pathological *β* oscillations, we first identified the main structures generating *β* resonances (Section 2.1). Following we studied the effects of dopamine depletion on the two independent *β* oscillators (Section 2.2). Then, we focused on the consequences of their interplay (Section 2.3) and investigated the role of dopamine depletion in shaping the spectral dynamics of the system (Section 2.4). Finally, in Section 2.5 we validated our model against recent experimental reports on the role of cortical inputs, STN and GPe activity in the generation of pathological *β* oscillations.

### 2.1 Identification and characterization of *β* oscillators

**Fig 3:**
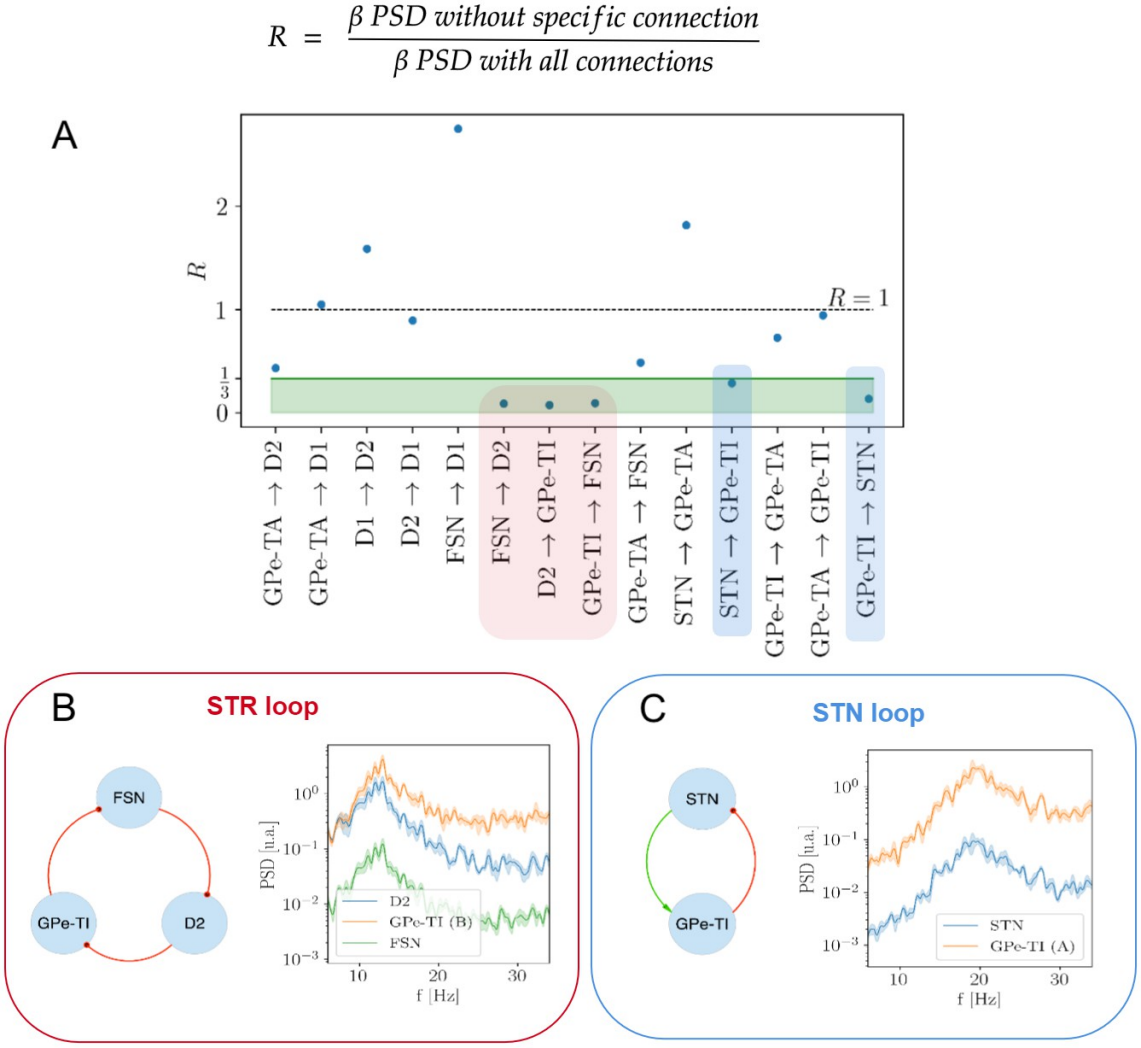
Identification of most relevant oscillators and investigation of their spectral properties. **(A)** Rationale about the selection of salient nodes in the BG network for a single simulation run. The nodes, taken together, define the STR and the STN loops (in blue and red shadow respectively). **(B)** STR loop with FSN, GPe-TI and D2 nuclei, connected with feedforward inhibitory projections (in red) and with their natural mode. **(C)** STN loop with STN and GPe-TI nuclei, connected with excitatory feedforward (in green) and inhibitory feedback projections (in red) and their natural mode.

In order to identify the connections mostly contributing to the generation of *β* oscillations, we considered the Complete Model of the BG (see Fig 1 and Methods Section 1.1) and computed the residual *β* power *R*(*c*) associated with the removal of each connection *c* from the network (see 1.3 for details). According to the definition in Eq (1), the more relevant connections are expected to be associated with lower values of the ratio *R*, hence we fixed a threshold $\bar{R}=\frac{1}{3}$ and focused on the connections with $R<\bar{R}$. As a result, five connections were identified as main responsible for the onset of *β* activity (Fig 3A): FSN→D2, D2→GPe-TI, GPE-TI→FSN, STN→GPe-TI and GPe-TI→STN. These connections set up two distinct oscillators, one involving FSN, D2 and GPe-TI nuclei (STR loop, Fig 3B) and the other involving STN and GPe-TI nuclei (STN loop, Fig 3C). The spectral analyses of the activity of the two oscillators (isolated from the other nuclei by setting *ε* = 0, see Methods) showed that:

$$\begin{array}{c} \begin{array}{cc} & f_{\text{STN}}=\left(18.9\pm 0.3\right)\;\text{Hz}\\ & f_{\text{GPe-TI}\;\text{(A)}}=\left(19.1\pm 0.2\right)\;\text{Hz} \end{array}\;\;\;\begin{array}{cc} & f_{\text{D2}}=\left(13.1\pm 0.2\right)\;\text{Hz}\\ & f_{\text{GPeTI}\;\text{(B)}}=\left(13.6\pm 0.3\right)\;\text{Hz}\\ & f_{\text{FSN}}=\left(13.3\pm 0.2\right)\;\text{Hz} \end{array} \end{array}$$ (6)

hence the STR loop has a proper frequency *f*_STR_ ∼ 13 Hz while STN naturally oscillates at *f*_STN_ ∼ 19 Hz.

### 2.2 Effect of dopamine depletion on the independent two main *β* oscillators

**Fig 4:**
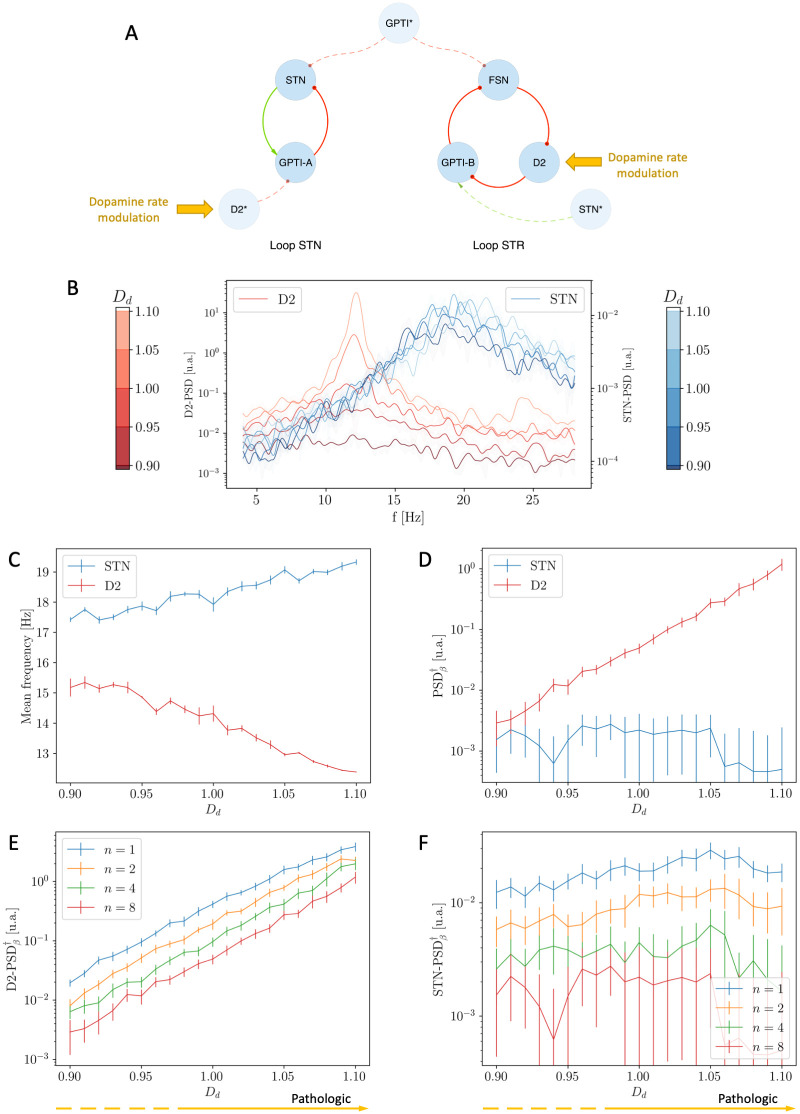
Effects of Dopamine Depletion *D*_*d*_ on the two independent oscillators. **(A)** Schematic representation of the Simplified Model in the non-interacting case. **(B)** Mean frequencies of STN (blue) and D2 (red) nuclei as a function of dopamine modulation *D*_*d*_: note that the two mean frequencies do NOT converge due to the increase of *D*_*d*_. **(C)** Unbiased measure of the intensity of *β* activity as a function of *D*_*d*_: note that the intensity of the D2 resonance grows for increasing values of *D*_*d*_. **(D-E)** Unbiased measure of the intensity of *β* activity in D2 (D) and STN (E) as a function of *D*_*d*_ and for different sizes *n* of the populations.

In this section we analyse the effects of dopamine depletion on the two separate, independent *β* oscillators. In order to do so, we employ the Simplified Model (see Methods Section 1.4 and Fig 4A), fix the value of *ε* = 0.00 and study the behaviour of the system as a function of the *D*_*d*_ parameter, which regulates the severity of dopamine depletion. Varying *D*_*d*_ led to different spectral modulations in the two oscillators (Fig 4B): the mean frequency of the STN nucleus remains close to ∼ 19 Hz, while that of the D2 population moves from ∼ 16 Hz, that is the mean value between 8 and 24 (see Methods and Eq (4)), to the natural frequency of the D2 oscillator at ∼ 13 Hz (Fig 4C); since the two oscillators do not interact, the system does not undergo any process of synchronization.

The intensity of *β* activity in the STN loop, measured through the STN nucleus, is only slightly affected by the variation of *D*_*d*_; conversely, the intensity of *β* activity in the STR oscillator, measured through the D2 nucleus, strongly increases with *D*_*d*_ (Fig 4C). Crucially, however, the intensity of these oscillations does not persist in the STN (Fig 4D) loop nor in the STR one in the limit of large populations (Fig 4E). This suggests that the increase of *β* activity in the isolated loops is not sufficient to account for the emergence of the pathological oscillations in realistic conditions.

### 2.3 Loops interplay

The results in the previous sections showed that as the value of *D*_*d*_ is increased, the STR oscillator increases its power. As a consequence, both the STN and STR loops may contribute to the generation of *β* oscillations in this condition. Moreover, since both loops contain the GPe-TI population, the two oscillators may interact with one another: the objective of this section is the analysis of the consequences of this interplay.

We now consider the Simplified Model, fix the value of *D*_*d*_ = 1.00 and analyse the effects of increasing the value of the *ε* parameter which regulates the intensity of the connections between the two oscillators (see Methods Section 1.4).

**Fig 5:**
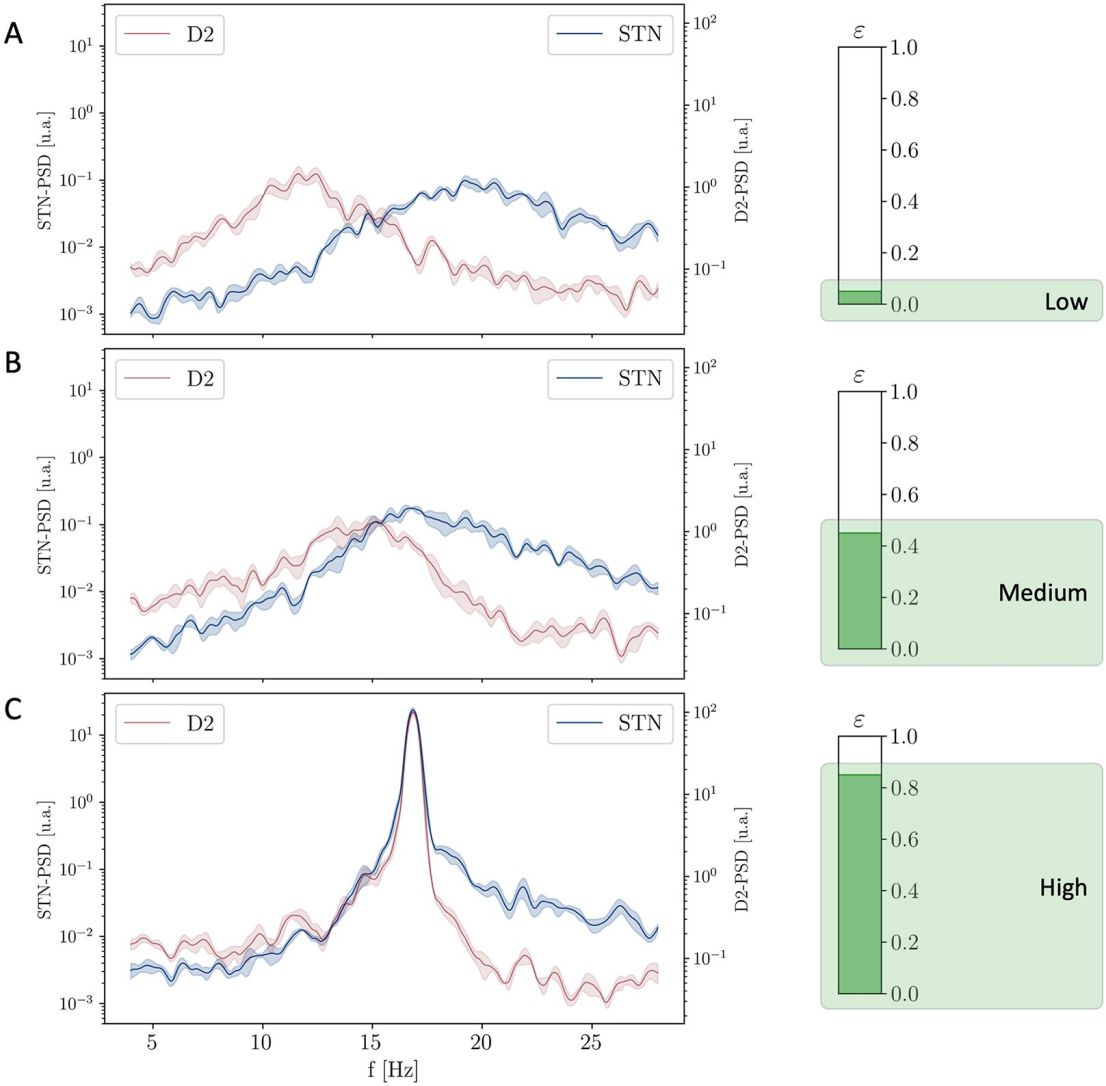
Evolution of the PSD of the STN and D2 populations for increasing values of the coupling strength *ε*. **(A)** PSD of STN and D2 nuclei related to a low level of coupling (*ε* ∼ 0.05) showing asynchronous states. **(B)** PSD of both STN and D2 nuclei in relation with an intermediate level of coupling (*ε* ∼ 0.45) showing larger peaks increasingly including the intermediate region. **(C)** PSD of STN and D2 nuclei related to a high level of coupling (*ε* ∼ 0.85) showing the emergence of a common oscillatory mode.

Interplay variability resulted in fundamental changes in the spectral dynamics of the system (Fig 5): for very low values of the coupling *ε* the STR and STN loops (measured through the D2 and STN nuclei respectively) exhibit a power spectrum which is not dissimilar from the case of the independent oscillators (*ε* = 0, *D*_*d*_ = 1.00) (Fig 3B and 3C). As the value of the coupling *ε* is increased, the PSDs of both populations become affected by the presence of the other resonance and present a broader peak which increasingly include the intermediate region between the natural frequencies of the two independent oscillators. Finally, for high values of *ε* ∼ 0.85, the oscillators complete their process of synchronization: the two separate resonances disappear and a single prominent peak emerges at an intermediate frequency.

**Fig 6:**
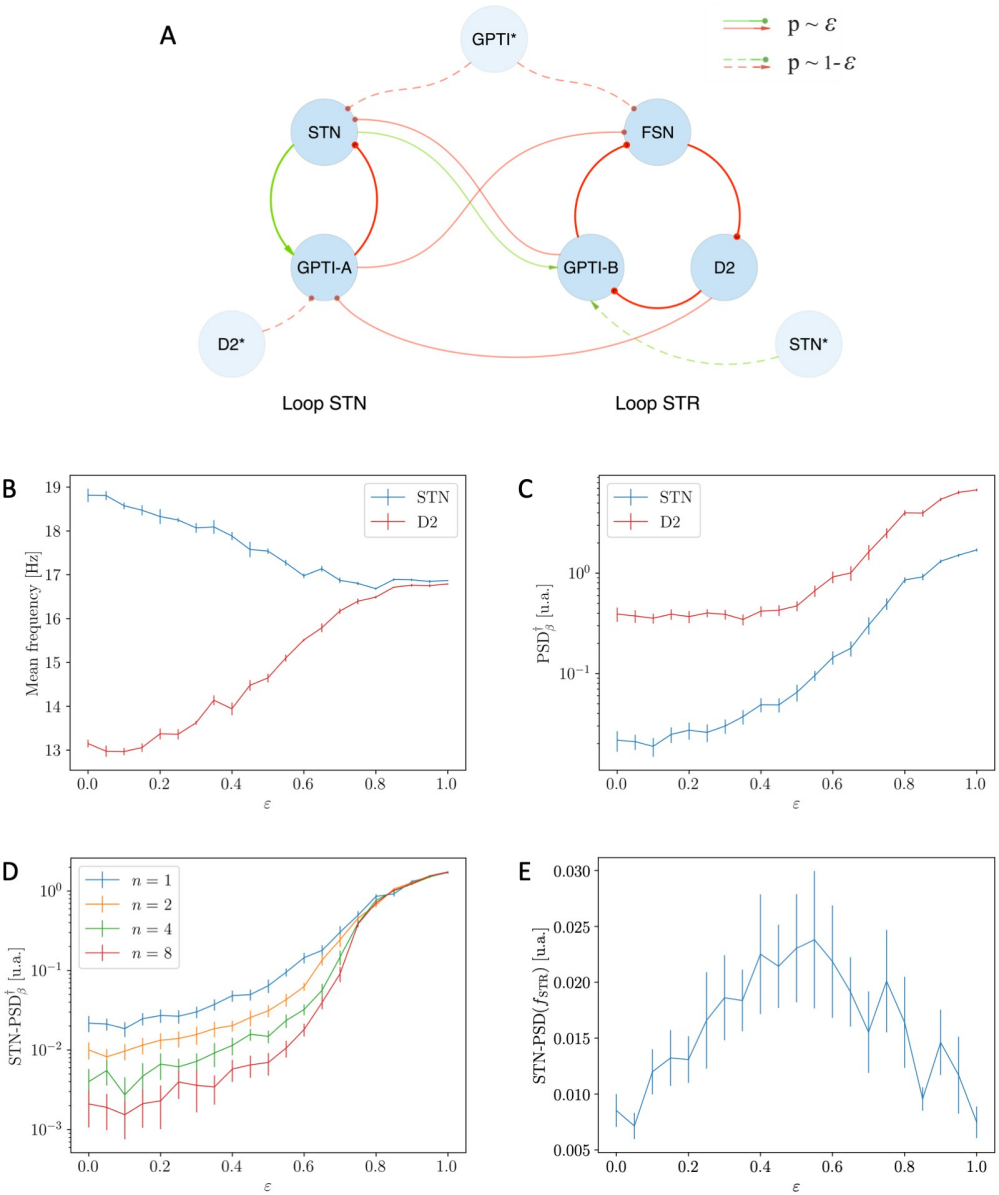
Effects of the coupling strength *ε* on the Simplified Model. **(A)** Schematic representation of the Simplified Model: the modulation of the coupling strength between the STR and STN loops is obtained by varying the connection probabilities *p*(*ε*, *S* → *T*) = *εp*_1,*S*→*T*_ (see Eq (2)) of *inter*-loops connections. **(B)** Mean frequencies of STN (blue) and D2 (red) nuclei as function of the coupling strength *ε*: the two mean frequencies converge due to the increase of the coupling strength between the two oscillators. **(C)** Unbiased measure of the intensity of *β* activity as a function of *ε*: note the remarkable growth of the intensity in the synchronous regime. **(D)** Unbiased measure of the intensity of the STN *β* activity as a function of *ε* for different values of the population size *n*: the intensity of *β* activity is preserved only in the synchronous regime. **(E)** Value of the STN-PSD nuclei computed at the natural frequency of the STR oscillator as a function of the coupling strength. These results are associated with the analogous shown in Fig A in S1 Text.

In order to highlight this behaviour, we computed the average frequency of the STR and STN loops (with specific measurements respectively at the D2 and STN nuclei) as a function of *ε*. As expected, the two frequencies start from the natural frequencies of the two oscillators (∼ 13 Hz and ∼ 19 Hz in the region of *ε* = 0) and, as the interplay between the two loops increases, converges to an intermediate common frequency *f* ∼ 16 Hz (*ε* = 1, see Fig 6B). Even more interestingly, the process of synchronization is associated with a strong increase in the intensity of the activity in the *β* regime (Fig 6C).

In order to verify whether the properties highlighted above persist in the limit of large populations, we repeated the analysis with the size of each population multiplied by a factor *n* ∈ [2, 4, 8] (see Methods sec 1.8). The results of this analysis highlight two different regimes (see Figs 6D and A in S1 Text):

- in case of low synchronization (*ε* ∼ 0) the intensity of *β* oscillations goes to zero in the large *n* limit;
- in case of high synchronization (*ε* ∼ 1) the intensity of *β* activity is preserved.

On the one hand, these results confirm that the establishment of a state of complete synchronization and the consequent onset of prominent *β* activity in the model are stable for variations in populations size; on the other hand, in accordance with the results in the previous section, they suggest that single loop models are not capable of explaining strong *β* activity in the limit of large populations.

A further insight on the process of synchronization is finally captured by the value of PSD_STN_(*f*_STR_) (i.e., the power spectral density of the STN population computed at the natural frequency of the STR loop; Fig 6E). Particularly, for low coupling, this value is low as STN uniquely oscillates at the natural frequency of the STN loop (Fig 5A). In the intermediate regime (*ε* ∼ 0.5) higher values are registered since the activity of the STN nucleus becomes more affected by the STR oscillator (Fig 5B). Finally, for *ε* ∼ 1, the value of PSD_STN_(*f*_STR_) decreases again as the two oscillators synchronize to a novel frequency which is different from both the natural ones (Fig 5C). A very similar trend is shown by the power spectral density of the D2 population computed at the natural frequency of the STN loop (see Fig A in S1 Text).

### 2.4 Role of dopamine in *β* synchronization dynamics

In the previous section, we described the effects of synchronization as a result of increasing the value of the coupling *ε* between the two *β* oscillators. In the real network, however, the value of *ε* is fixed. This fact implies that there must be something else, within the network, which is responsible for the increase of the interaction between the two oscillators and hence drives the system towards higher degrees of synchronization. Similarly to Section 2.2 we now focus on the effects of increasing the severity of the condition of dopamine depletion by regulating the value of *D*_*d*_; however, differently from there, we now study the effects of the variation of *D*_*d*_ when interplay between the two oscillators is present.

#### Role of dopamine in the Simplified Model

**Fig 7:**
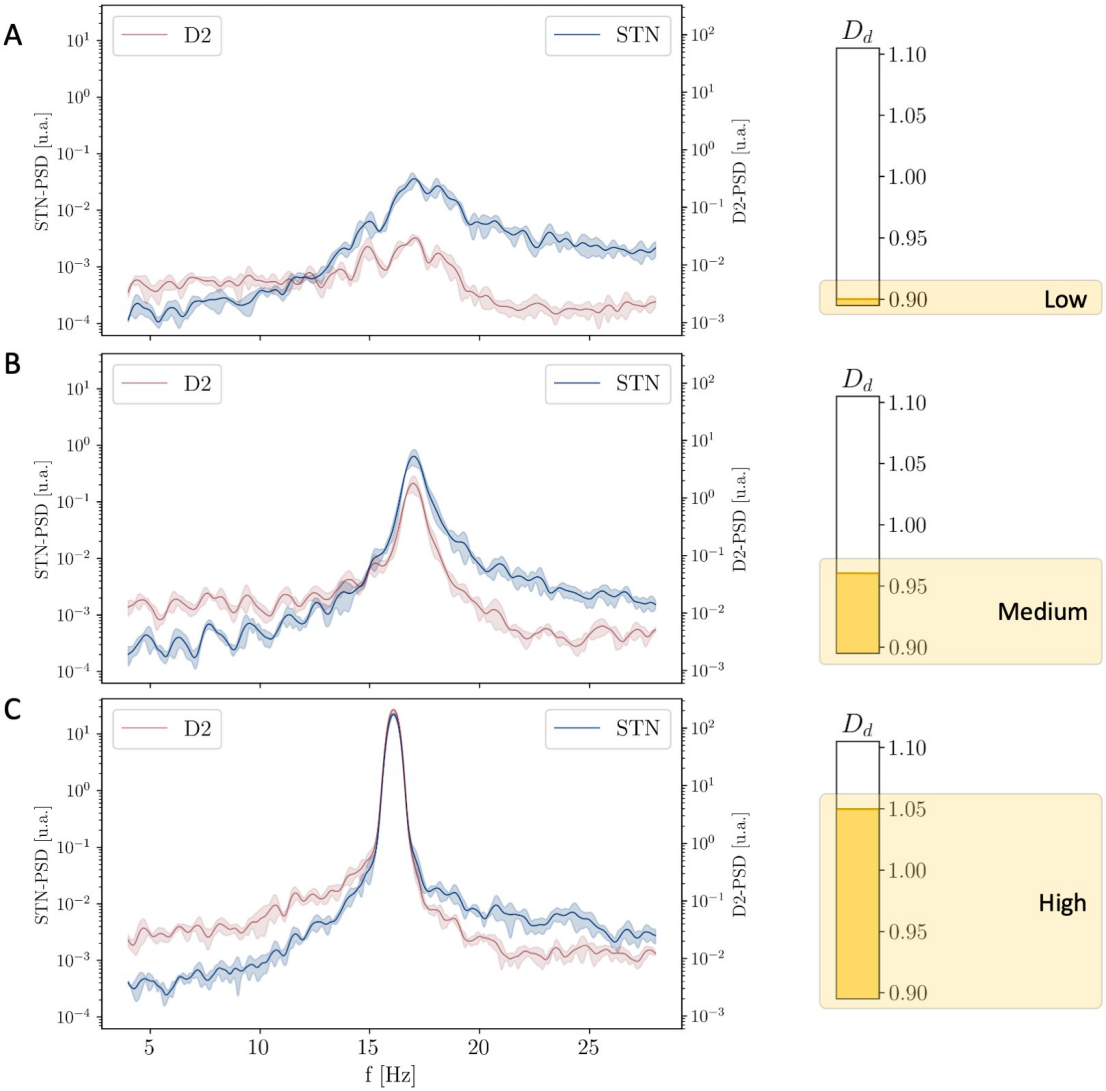
Evolution of the PSD of the STN and D2 populations for increasing values of Dopamine Depletion *D*_*d*_. PSD of both STN and D2 nuclei related to low **(A)**, intermediate **(B)** and high **(C)** levels of dopamine depletion (*D*_*d*_ = 0.9, 0.97 and 1.05 respectively): for low values of *D*_*d*_ the system is characterized by the only STN-loop resonance; the increase of *D*_*d*_ leads to higher degrees of interaction and to the emergence of a unique resonance at an intermediate frequency.

We start considering the Simplified Model and introduce the coupling between the two oscillators by setting the value of *ε* = 0.75. Similarly to the case in Section 2.2, the increase of *D*_*d*_ determines the increase of the intensity of *β* activity in the STR loop. Crucially however, in this case, the increase in the intensity of the STR Loop leads the two oscillators to undergo a process of synchronization: for low values of *D*_*d*_ the two loops interact poorly (the STR oscillator is almost absent and only the STN resonance at ∼19 Hz characterizes the status of the system; see Fig 7A); as the value of *D*_*d*_ is increased, the enhancement in the intensity of the STR oscillator implies higher degrees of interaction between the two loops and consequently leads to the emergence of a single resonance at an intermediate frequency (∼ 16 Hz; see Fig 7B and 7C).

**Fig 8:**
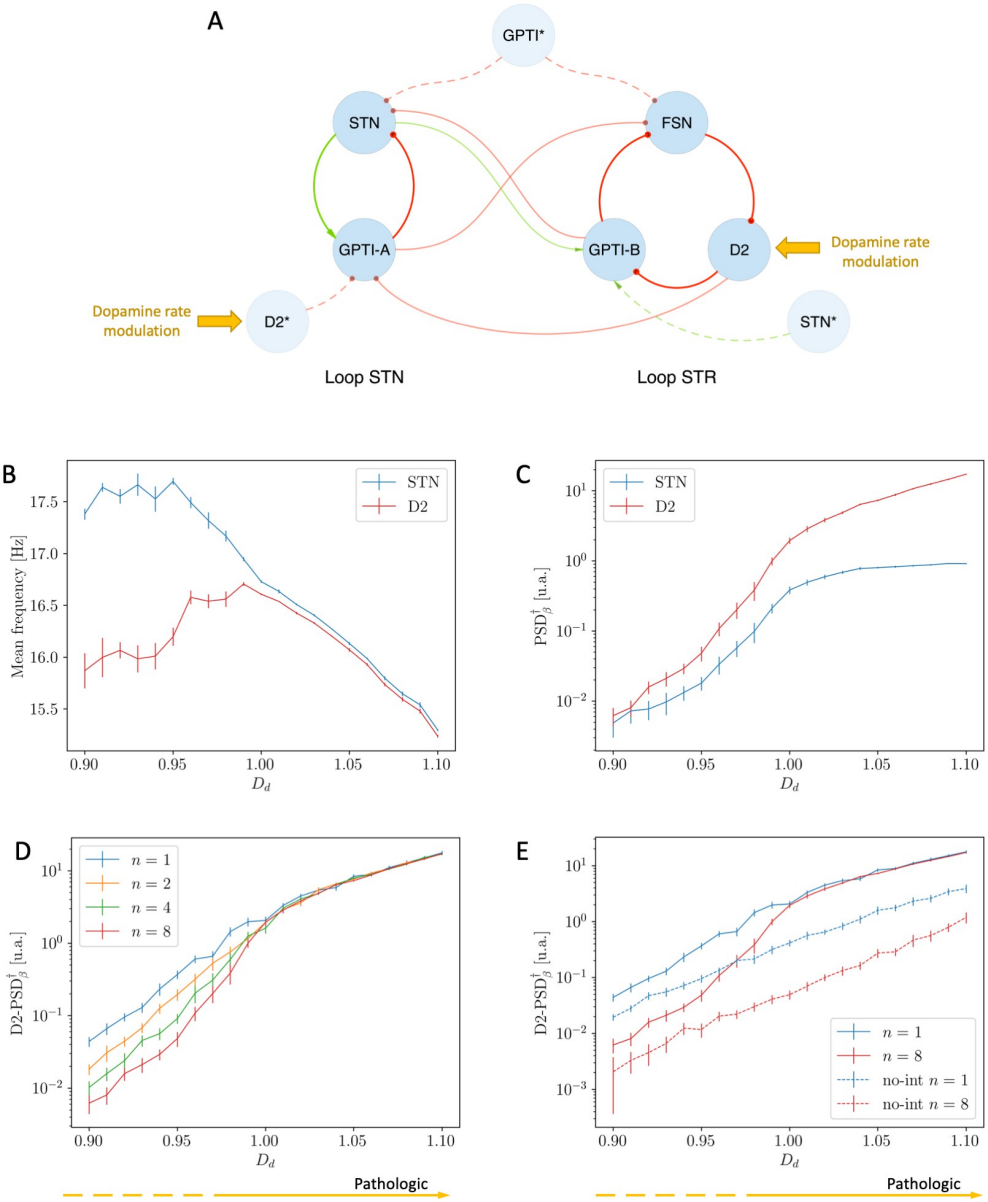
Effects of Dopamine Depletion *D*_*d*_ on the Simplified Model (*ε* = 0.75). **(A)** Schematic representation of the Simplified model in the interacting case: the targets of Dopamine modulation are highlighted. **(B)** Mean frequencies of STN (blue) and D2 (red) nuclei as function of dopamine modulation *D*_*d*_: the two mean frequencies converge due to the increase of *D*_*d*_. **(C)** Unbiased measure of the intensity of *β* activity as a function of *D*_*d*_: note the remarkable growth of the intensity in the synchronous regime. **(D)** Unbiased measure of the intensity of the D2 *β* activity as a function of *D*_*d*_ for different values of the population size *n*: the intensity of *β* activity is preserved only in the synchronous regime. **(E)** Unbiased measure of the intensity of the D2 *β* activity as a function of *D*_*d*_ for *n* = 1 and *n* = 8 and comparison between the interacting (*ε* = 0.75, continuous lines) and non-interacting condition (*ε* = 0.00, dashed lines): the intensity of *β* activity is preserved if and only if the two oscillators are synchronized. These results are associated with the analogous shown in Fig B in S1 Text.

Similarly to the increase of *ε* (Section 2.3), the increase of *D*_*d*_ determines the convergence of the mean frequencies of oscillation of the STN and D2 populations (Fig 8B) and a remarkable increase in the intensity of the pathological oscillations (Fig 8C).

Further, the analysis of the limit of large populations shows that:

- if *ε* = 0.75 and *D*_*d*_ ∼ 1.1 (synchronized oscillators), the intensity of *β* activity is preserved (Figs 8D and B in S1 Text);
- if *ε* = 0.75 and *D*_*d*_ ∼ 0.9 (non-synchronized oscillators), the intensity of *β* activity decreases (Figs 8D and B in S1 Text);
- if *ε* = 0.00, for any value of *D*_*d*_ (non-synchronized oscillators), the intensity of *β* activity decreases (Figs 8E and B in S1 Text).

In accordance with previous results, this proves that the intensity of *β* activity in the large *n* limit is only preserved when the two oscillators are in the synchronous state (*ε* = 0.75 and *D*_*d*_ ∼ 1.1).

Beside that, the comparison between the uncoupled (*ε* = 0.00) and coupled (*ε* = 0.75) cases highlights that the increase in the intensity of *β* activity is stronger in the latter case (Fig 8E).

The results proposed in this section concerning the role of dopamine in the case of the Simplified Model showed interesting changes in the functional and spectral dynamics of the two loops. For low values of *D*_*d*_, we observed a weak synchronisation between the STR and STN loops, which then oscillate around their natural resonance. Conversely, the more severe is the condition of dopamine depletion, the stronger the state of synchronisation of the two loops, and the more the activity of the network is characterized by a single, prominent oscillatory mode in the *β* regime.

#### Role of dopamine in the Complete Model

**Fig 9:**
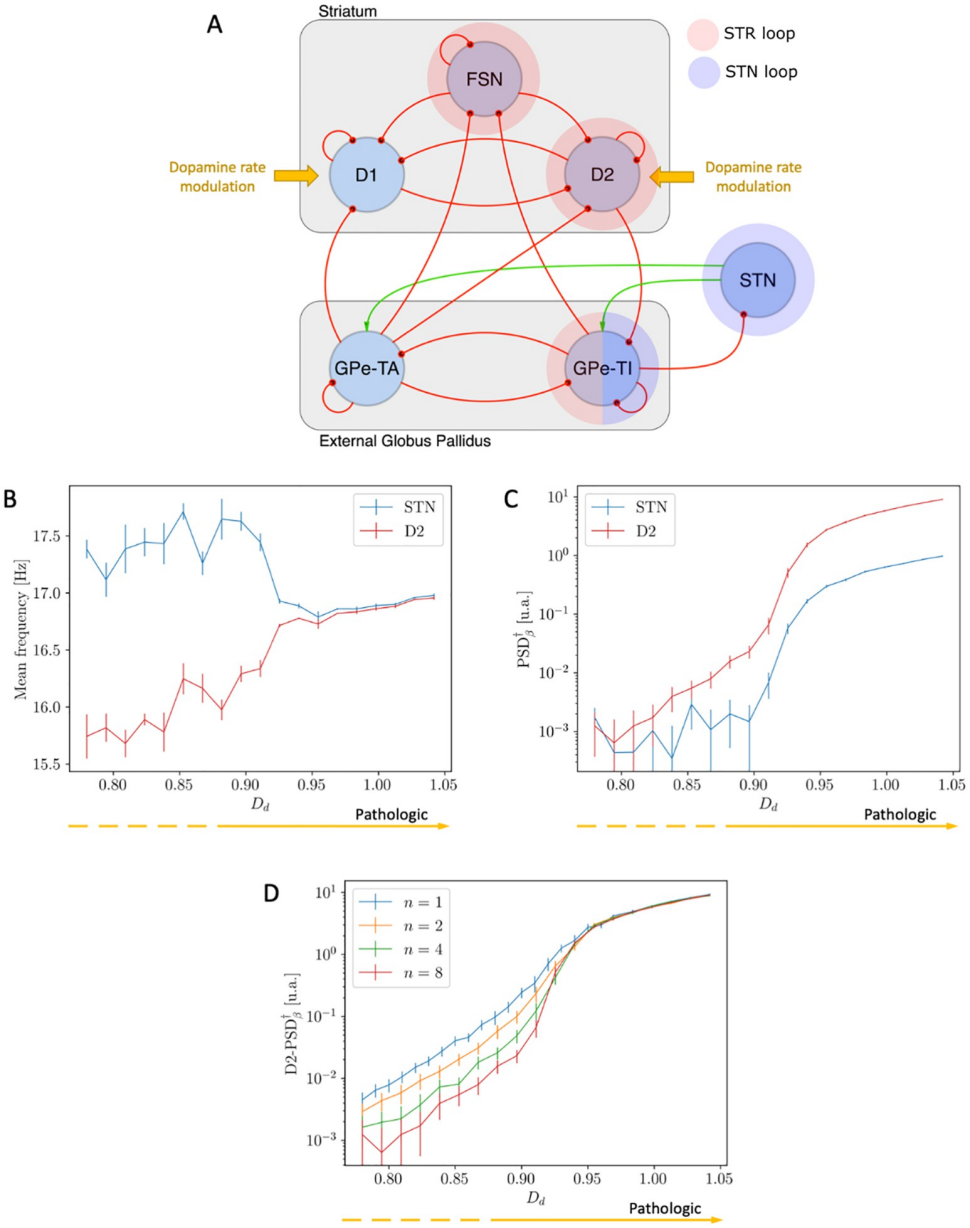
Effects of Dopamine Depletion *D*_*d*_ on the Complete Model. **(A)** Schematic representation of the Complete Model: the targets of Dopamine modulation are highlighted. **(B)** Mean frequencies of STN (blue) and D2 (red) nuclei as function of dopamine modulation *D*_*d*_: the two mean frequencies converge due to the increase of *D*_*d*_. **(C)** Unbiased measure of the intensity of *β* activity as a function of *D*_*d*_: note the remarkable growth of the intensity in the synchronous regime. **(D)** Unbiased measure of the intensity of the D2 *β* activity as a function of *D*_*d*_ for different values of the population size *n*: the intensity of *β* activity is preserved only in the synchronous regime. Analogous results about STN *β* activity are shown in Fig C in S1 Text.

In all previous analyses we focused on the properties of the emergent *β* activity in the context of the Simplified Model. In that case, we showed that the effects of dopamine depletion and the consequent process of synchronization of the two oscillators set up by the STR and STN loops play a major role in the onset of pathological, prominent *β* activity. In line with those results, in this section we will show that all the highlighted properties are preserved in the Complete Model of the BG (see Fig 9A). Particularly, even in this model, as the value of *D*_*d*_ is increased, the mean frequencies of the D2 and STN populations converge (Fig 9B) and the intensity of *β* oscillations increases and presents the characteristic speed up due to completion of the synchronization process (Fig 9C). Note that these behaviours and the onset of synchronization associated with dopamine depletion, which represent the key result of our work, are robust to alterations in network and single neurons parameters (see Fig E in S1 Text).

Finally, in accordance with previous results, the size analysis confirms that our model is capable of capturing prominent *β* activity also in the limit of large populations (see Figs 9D and C in S1 Text).

Please note that in all the studies regarding the effects of dopamine depletion (Figs 4, 7, 8B, 8C, 9B and 9C) we showed the results related to the case *n* = 8. This has been necessary to capture the relevant properties of the system in a clearer way (for example, the reader can compare the speed up in the increase of *β* activity in case of *n* = 1 and *n* = 8 shown in Fig 8E). This choice does not affect the results of the large *n* limit analysis.

#### *β* bursting activity

The presented results have been obtained by mediating over different time intervals and different simulations (see Methods Section 1.7). Performing this operation denied us the possibility of investigating the transient properties of the network.

**Fig 10:**
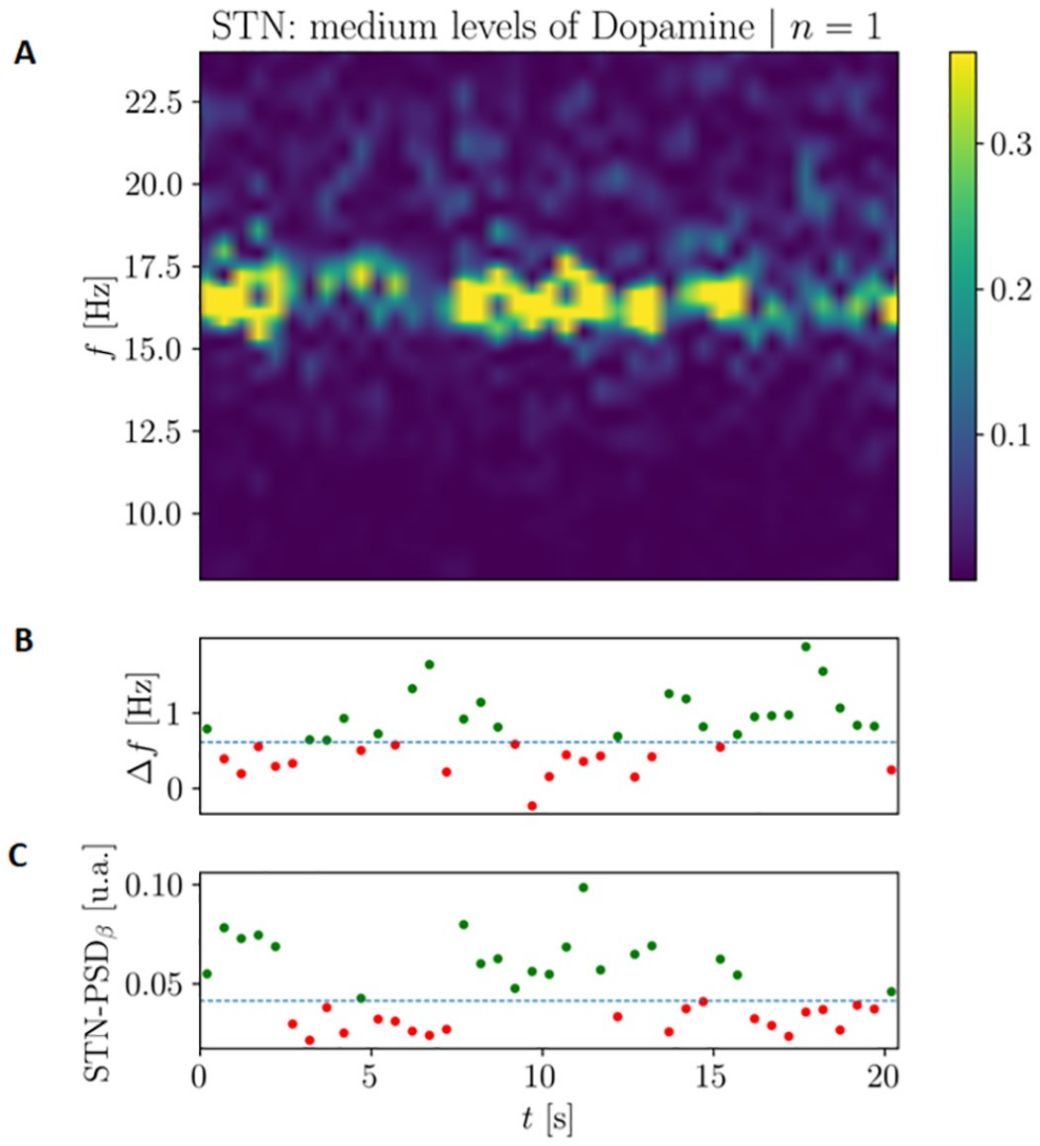
Bursting dynamics for intermediate values of dopamine (*D*_*d*_ = 0.90) in the case of the Complete Model of the BG. **(A)** Periodogram of the STN population activity highlighting the bursting characterization along a period of 20 seconds. **(B)** Difference between the instantaneous mean frequency of STN and D2 nuclei. **(C)** Instantaneous values of PSD for the STN nucleus. The dashed lines in the inferior subplots represent the median values of Δ*f* and *β* STN Mean PSD respectively.

One of the most important feature of pathological *β* activity in the BG is that it presents a strongly phasic characterization [20, 21]. As shown in Fig 10, our model is capable of capturing this characterization. In particular, for intermediate values of *D*_*d*_ (*D*_*d*_ = 0.90), the system gains temporary access to the synchronous condition and the two oscillators continuously switch between the synchronous and asynchronous states. Moreover, when the value of Δ*f*(*t*) = *f*_STN_(*t*) − *f*_D2_(*t*) decreases (meaning that the system spends more time in the synchronous status), the intensity of the *β* oscillations increases (see the red-green and green-red matching of points related to the same time intervals in the two inferior subplots of Fig 10). Analyzing the distribution of high *β* power intervals in 10 s long simulations for different levels of *D*_*d*_ we found strong differences in the skewness between values between high and low levels of *D*_*d*_ (see Fig F in S1 Text). For low values of *D*_*d*_ the *β* power distribution displays a long right tail (skewness between 0.85 and 1.5), indicating the presence of transient bursting. Therefore *β* bursts, although uncommon, are possible, even for low levels of dopamine depletion. On the other hand, when the synchronization occurs, the intensity of *β* activity increases and the distribution does not show any tail (skewness between -0.29 and 0.29). In this condition, high *β* power intensity is stable and not transient anymore. These results are in accordance with the previous considerations and indicate once again that the more the two oscillators interact, the more the system spends time in the synchronous state and the higher is the intensity of *β* activity within the network.

### 2.5 Comparison with relevant experimental results on the role of specific nuclei in pathological *β* oscillations

**Fig 11:**
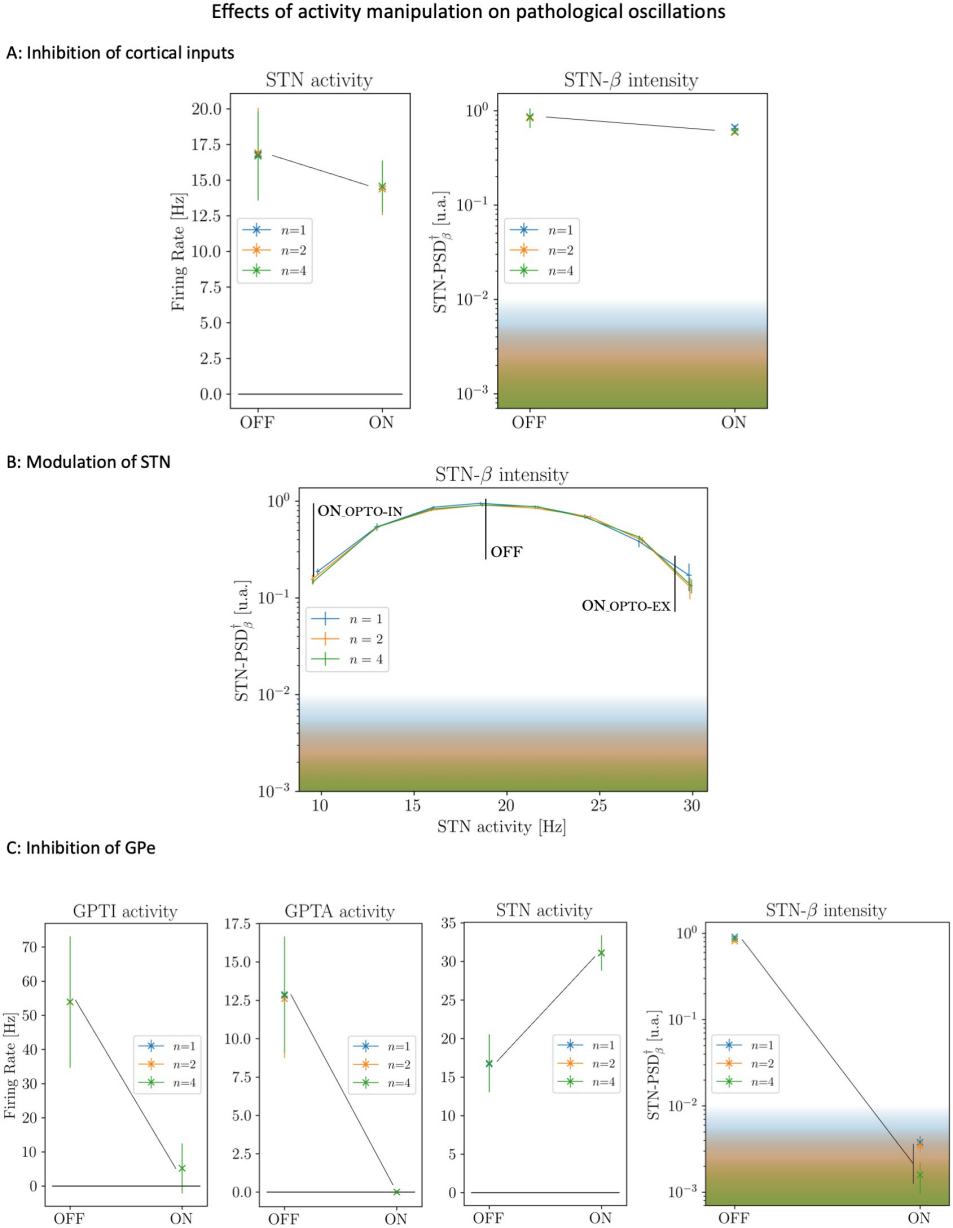
Effects of activity manipulations on network dynamics. All manipulations are based on the opto-genetic stimulations discussed in [49]. OFF values are measured in pathologic conditions (Complete Model with *D*_*d*_ = 1.03) while ON values are measured when the opto-genetic perturbations are applied. **(A)** Simulated effects of motor cortex opto-inhibition on STN discharge rate (left) and intensity of *β* oscillations (right) (see also Figs 1k-l and 1i-j in [49]). **(B)** Predictions of the model on the intensity of *β* activity as a consequence of large variations of the STN discharge rate (see also Figs 2e-f-g and 5i-k in [49]). **(C)** Consequences of GPe opto-inhibition on GPe-TI and GPe-TA nuclei (first panels on the right) and predictions of the model on the effects of GPe opto-inhibition on STN discharge rate and intensity of *β* oscillations (right panels) (see also Figs 4k-l and 4i-j in [49]). Shaded areas in the plots reporting *β* intensity correspond to the values of ${\text{STN-PSD}}_{\beta }^{†}$ observed for low *D*_*d*_ in the Complete Model (see Fig C in S1 Text).

The results in the previous sections support the hypothesis that pathological *β* oscillations originate from the synchronization of a striatopallidal loop and a subthalamic-pallidal loop mediated by the GPe. The relevance of GPe in the generation of *β* oscillations was recently proved by a work of Mallet group [49]. We wondered then to which extent our model was able to account for the experimental results found in this work. First, in [49] the cortical origin of *β* oscillations is ruled out by observing that optogenic cortical silencing led to a decrease of STN activity but did not significantly affect *β* power. To test whether our model was coherent with these results, we simulated the partial suppression of cortical inputs as described in Methods Section 1.6. Introducing this perturbation in our model, we found that the inhibition of inputs from motor cortex did entail a decrease (∼ −15%) in the firing rate of STN (Fig 11A); but did not suppress pathological *β* activity (Fig 11A) in line with the results in [49] (see their Fig 1i-j and 1k-l respectively). Interestingly, the same study showed that both suppression (see Fig 2 in [49]) and amplification (see Fig 5 in [49]) of STN activity have a small effect on *β* intensity, suggesting that STN might only have, in the words of the authors, “a supportive role” in the generation of pathological *β* oscillations. In order to simulate these STN activity manipulations, we induced a modulation of STN activity as described in Methods Section 1.6. Coherently with [49], in our model the intensity of *β* oscillations decreased when STN activity moved away from its reference value (∼ 18 Hz), but remained one order of magnitude stronger than the values observed for low *D*_*d*_ even for relatively large variations of the STN discharge rate (Fig 11B). Crucially, in [49] opto-inhibition of GPe induced an increase in STN activity but at the same time a complete suppression of pathological *β* oscillations. We simulated the inhibition of GPe as described in Methods Section 1.6. In this condition, the model displayed a significant increase in STN activity (∼ +85%) and the complete suppression of pathological *β* oscillations (Fig 11C), coherently with what observed in [49] (see Figs 4i-j and 4k-l respectively).

Overall, these results shows that the model is capable of reproducing the main results about the role of the different nuclei in the generation of pathological *β* oscillations displayed in [49].

## Discussion

We characterized the interplay between two loops generating *β* oscillations within the BG: one between GPe and striatal populations FSN and D2 and one between GPe and STN. While for high values of dopamine the two loops are both weak and act largely as independent, when dopamine is depleted the striatopallidal loop increases its strength, and this leads to the synchronization and mutual reinforcement between the two loops and eventually to strong oscillations in the whole BG network. We suggest that this mechanism plays a key role in the generation of abnormal *β* oscillations in PD. D2 neurons are known to increase their firing activity due to the loss of inhibitory effect of dopamine and to strongly synchronize in the *β* range in dopamine-depleted conditions [14]. Our modeling work highlights a mechanism in which the former phenomena leads to the latter due to the internal resonances of the BG network.

As a strong evidence of our hypothesis, we showed that our model is able to account for main results in [49], and to shed light on the underlying mechanisms. To date, no computational model was able to comprehensively explain the observed dynamics: first of all, such results can not be accounted for by models based on a cortical origin of *β* oscillations; further, in the Discussion of [49] the authors mention two computational models suggesting the importance of increased striatal inhibition to induce abnormal oscillations in the GPe-STN network [35, 83], but stress that these models did not clarify the mechanism through which this increase leads to the onset of synchronization at *β*-frequency. In contrast, the computational model that is proposed in this study succeeds in giving a causal reason for the generation of pathological oscillations by considering the interaction between the STR and STN loops under dopamine depleted condition.

Our results are coherent with classic results highlighting the role of the STN-GPe loop in generating *β* oscillations [27] as this loop indeed exists and does play an important role. The key difference is that in our view this loop is not simply modulated by the intensity of the striatal inhibition to GPe, but rather by the intensity of the *β* oscillations of the striatopallidal loop. In a relevant work, Corbit and colleagues introduced a detailed small scale, closed-loop model of the striatopallidal loop [57] and showed how it generated *β* oscillations. Later models, including [58] which was the model laying the ground for this work, implemented then this loop in their network descriptions ([84, 85]), but did not explicitly address how the two loops interacted in originating *β* oscillations, rather focusing on action selection. Interestingly, a recent study by the same group focusing on transient responses of the BG network [86] also captured *β* activity, but did not address the question of its origins. A key paper, [87] proposes that *β* oscillations rely on both the STN-GPe loop and the cortex (rather than exclusively on the STN-GPe loop). One limitation of our model is indeed that it neglects the effect of temporal evolution of cortical inputs, as the presence of cortical *β* bursts or input correlations, which might contribute to the development of *β* oscillations [15]. Moreover, our model is currently focused on account for experimental findings on rats, hence translation to primates and humans is nontrivial. A third limitation is that we only considered effects of dopamine in D1 and D2 activity, while connectivity changes might play a role in *β* power modulation (see Fig. S5E and the interesting pre-print [88]). Further limitations of our model include the lack of dynamics to account for plasticity effects, and the fact that selecting an adaptive integrate and fire neuron model we did not take into account specific currents that are included in Hodgkin and Huxley models of basal ganglia (e.g. [89]).

As a final remark, the presented size analyses highlight that the intensity of *β* activity is preserved when the two oscillators are in the synchronous regime. On the one hand this confirms that our model is capable of reproducing *β* activity also in the limit of large populations; on the other hand it suggests that single loop models (e.g. [35, 39, 57]) may not be capable of explaining prominent *β* activity.

In future works we will investigate how our model of the mechanisms underlying the pathological oscillations in PD can help in the understanding of the interplay between therapies for PD and *β* activity. Dopamine agonists are known to alter *β* activity in the BG [90, 91]. In our model, *D*_*d*_ values could both increase due to the progression of PD and decrease due to the action of dopamine agonists, which would be coherent with the decrease in *β* activity induced by drugs [92]. However, to proper model this aspect we would have to take into account other pathophysiological aspects [93, 94].

Moreover, it would be interesting to simulate the action of DBS as in [95–98] also in our model to evaluate its effects on suppressing *β* oscillations and compare these to experimental results [99–101]. Recent studies highlighted a dynamical and functional distinction between a low *β* range [12–20] Hz and a high *β* range [20–30] Hz [102–104]. Even if the *β* activity discussed in the present work is within the low range, we do not claim to have specifically captured the dynamics of this band. Understanding the different mechanisms underlying low and high *β* could also shed light on the underpinnings of PD therapies, as both dopamine agonist [105] and DBS [106] seem to have a specific effect on the low *β* range.

## Supporting information

S1 Text Supporting information. This file collects the following supporting tables and figures: **Table A**. Parameter changes from the Complete to the Simplified Model. **Fig A**. Effects of the coupling strength *ε* on the Simplified Model: plots for the D2 nucleus. **Fig B**. Effects of Dopamine Depletion *D*_*d*_ on the Simplified Model (*ε* = 0.75): plots for the STN nucleus. **Fig C**. Effects of Dopamine Depletion *D*_*d*_ on the Complete Model: plot for the STN nucleus. **Fig D**. Effects of the connectivity alterations due to Dopamine Depletion on the intensity of *β* activity. **Fig E**. Robustness of the results to alterations of the connectivity parameters and neuron and population parameters. **Fig F**. Probability distribution of the instantaneous intensity of STN activity for different degrees of Dopamine Depletion.
(PDF)

## References

[1] Obeso JA, Rodriguez-Oroz MC, Stamelou M, Bhatia KP, Burn DJ. The expanding universe of disorders of the basal ganglia. The Lancet. 2014;384(9942):523–531. doi: 10.1016/S0140-6736(13)62418-6 24954674

[2] Bergman H, Horn A. The Hidden Life of the Basal Ganglia: At the Base of Brain and Mind. Brain. 2021.

[3] Feigin VL, Nichols E, Alam T, Bannick MS, Beghi E, Blake N, et al. Global, regional, and national burden of neurological disorders, 1990–2016: a systematic analysis for the Global Burden of Disease Study 2016. The Lancet Neurology. 2019;18(5):459–480. doi: 10.1016/S1474-4422(18)30499-X 30879893 PMC6459001

[4] Albin RL, Young AB, Penney JB. The functional anatomy of basal ganglia disorders. Trends in neurosciences. 1989;12(10):366–375. doi: 10.1016/0166-2236(89)90074-X 2479133

[5] Onofrj M, Thomas A. Acute akinesia in Parkinson disease. Neurology. 2005;64(7):1162–1169. doi: 10.1212/01.WNL.0000157058.17871.7B 15824341

[6] Berardelli A, Rothwell JC, Thompson PD, Hallett M. Pathophysiology of bradykinesia in Parkinson’s disease. Brain. 2001;124(11):2131–2146. doi: 10.1093/brain/124.11.2131 11673316

[7] Zimmermann R, Deuschl G, Hornig A, Schulte-Mönting J, Fuchs G, Lücking C. Tremors in Parkinson’s disease: symptom analysis and rating. Clinical neuropharmacology. 1994. doi: 10.1097/00002826-199408000-00001 9316677

[8] Perez-Lloret S, Negre-Pages L, Damier P, Delval A, Derkinderen P, Destée A, et al. Prevalence, determinants, and effect on quality of life of freezing of gait in Parkinson disease. JAMA neurology. 2014;71(7):884–890. doi: 10.1001/jamaneurol.2014.753 24839938

[9] Nutt JG, Bloem BR, Giladi N, Hallett M, Horak FB, Nieuwboer A. Freezing of gait: moving forward on a mysterious clinical phenomenon. The Lancet Neurology. 2011;10(8):734–744. doi: 10.1016/S1474-4422(11)70143-0 21777828 PMC7293393

[10] Nambu A. Seven problems on the basal ganglia. Current opinion in neurobiology. 2008;18(6):595–604. doi: 10.1016/j.conb.2008.11.001 19081243

[11] Mallet N, Ballion B, Le Moine C, Gonon F. Cortical inputs and GABA interneurons imbalance projection neurons in the striatum of parkinsonian rats. Journal of Neuroscience. 2006;26(14):3875–3884. doi: 10.1523/JNEUROSCI.4439-05.2006 16597742 PMC6674115

[12] McCarthy M, Moore-Kochlacs C, Gu X, Boyden E, Han X, Kopell N. Striatal origin of the pathologic beta oscillations in Parkinson’s disease. Proceedings of the National Academy of Sciences. 2011;108(28):11620–11625. doi: 10.1073/pnas.1107748108 21697509 PMC3136295

[13] Kondabolu K, Roberts EA, Bucklin M, McCarthy MM, Kopell N, Han X. Striatal cholinergic interneurons generate beta and gamma oscillations in the corticostriatal circuit and produce motor deficits. Proceedings of the National Academy of Sciences. 2016;113(22):E3159–E3168. doi: 10.1073/pnas.1605658113 27185924 PMC4896681

[14] Sharott A, Vinciati F, Nakamura KC, Magill PJ. A population of indirect pathway striatal projection neurons is selectively entrained to parkinsonian beta oscillations. Journal of Neuroscience. 2017;37(41):9977–9998. doi: 10.1523/JNEUROSCI.0658-17.2017 28847810 PMC5637121

[15] Manferlotti E, Vissani M, Mazzoni A, Kumar A. Correlated inputs to striatal population drive subthalamic nucleus hyper-synchronization. In: 2021 10th International IEEE/EMBS Conference on Neural Engineering (NER). IEEE; 2021. p. 255–258.

[16] Eisinger RS, Cagle JN, Opri E, Alcantara J, Cernera S, Foote KD, et al. Parkinsonian beta dynamics during rest and movement in the dorsal pallidum and subthalamic nucleus. Journal of Neuroscience. 2020;40(14):2859–2867. doi: 10.1523/JNEUROSCI.2113-19.2020 32107277 PMC7117906

[17] Valsky D, Blackwell KT, Tamir I, Eitan R, Bergman H, Israel Z. Real-time machine learning classification of pallidal borders during deep brain stimulation surgery. Journal of neural engineering. 2020;17(1):016021. doi: 10.1088/1741-2552/ab53ac 31675740

[18] Haumesser JK, Beck MH, Pellegrini F, Kühn J, Neumann WJ, Altschüler J, et al. Subthalamic beta oscillations correlate with dopaminergic degeneration in experimental parkinsonism. Experimental Neurology. 2021;335:113513. doi: 10.1016/j.expneurol.2020.113513 33148526

[19] Mallet N, Pogosyan A, Sharott A, Csicsvari J, Bolam JP, Brown P, et al. Disrupted dopamine transmission and the emergence of exaggerated beta oscillations in subthalamic nucleus and cerebral cortex. Journal of Neuroscience. 2008;28(18):4795–4806. doi: 10.1523/JNEUROSCI.0123-08.2008 18448656 PMC6670450

[20] Feingold J, Gibson DJ, DePasquale B, Graybiel AM. Bursts of beta oscillation differentiate postperformance activity in the striatum and motor cortex of monkeys performing movement tasks. Proceedings of the National Academy of Sciences. 2015;112(44):13687–13692. doi: 10.1073/pnas.1517629112 26460033 PMC4640760

[21] Murthy VN, Fetz EE. Oscillatory activity in sensorimotor cortex of awake monkeys: synchronization of local field potentials and relation to behavior. Journal of neurophysiology. 1996;76(6):3949–3967. doi: 10.1152/jn.1996.76.6.3949 8985892

[22] Deffains M, Iskhakova L, Katabi S, Israel Z, Bergman H. Longer *β* oscillatory episodes reliably identify pathological subthalamic activity in Parkinsonism. Movement Disorders. 2018;33(10):1609–1618. doi: 10.1002/mds.27418 30145811

[23] Mizrahi-Kliger AD, Kaplan A, Israel Z, Deffains M, Bergman H. Basal ganglia beta oscillations during sleep underlie Parkinsonian insomnia. Proceedings of the National Academy of Sciences. 2020;117(29):17359–17368. doi: 10.1073/pnas.2001560117 32636265 PMC7382242

[24] Mallet N, Pogosyan A, Márton LF, Bolam JP, Brown P, Magill PJ. Parkinsonian Beta Oscillations in the External Globus Pallidus and Their Relationship with Subthalamic Nucleus Activity. Journal of Neuroscience. 2008;28(52):14245–14258. doi: 10.1523/JNEUROSCI.4199-08.2008 19109506 PMC4243385

[25] Avila I, Parr-Brownlie LC, Brazhnik E, Castañeda E, Bergstrom DA, Walters JR. Beta frequency synchronization in basal ganglia output during rest and walk in a hemiparkinsonian rat. Experimental neurology. 2010;221(2):307–319. doi: 10.1016/j.expneurol.2009.11.016 19948166 PMC3384738

[26] Whalen TC, Willard AM, Rubin JE, Gittis AH. Delta oscillations are a robust biomarker of dopamine depletion severity and motor dysfunction in awake mice. Journal of neurophysiology. 2020;124(2):312–329. doi: 10.1152/jn.00158.2020 32579421 PMC7500379

[27] Plenz D, Kital ST. A basal ganglia pacemaker formed by the subthalamic nucleus and external globus pallidus. Nature. 1999;400(6745):677–682. doi: 10.1038/23281 10458164

[28] Bevan MD, Magill PJ, Terman D, Bolam JP, Wilson CJ. Move to the rhythm: oscillations in the subthalamic nucleus–external globus pallidus network. Trends in Neurosciences. 2002;25(10):525–531. doi: 10.1016/S0166-2236(02)02235-X 12220881

[29] Sato F, Lavallée P, Lévesque M, Parent A. Single-axon tracing study of neurons of the external segment of the globus pallidus in primate. Journal of Comparative Neurology. 2000;417(1):17–31. doi: 10.1002/(SICI)1096-9861(20000131)417:1<17::AID-CNE2>3.0.CO;2-I 10660885

[30] Hammond C, Bergman H, Brown P. Pathological synchronization in Parkinson’s disease: networks, models and treatments. Trends in neurosciences. 2007;30(7):357–364. doi: 10.1016/j.tins.2007.05.004 17532060

[31] Alavi SM, Mirzaei A, Valizadeh A, Ebrahimpour R. Excitatory deep brain stimulation quenches beta oscillations arising in a computational model of the subthalamo-pallidal loop. Scientific reports. 2022;12(1):1–20. doi: 10.1038/s41598-022-10084-4 35552409 PMC9098470

[32] Vitek JL, Zhang J, Hashimoto T, Russo GS, Baker KB. External pallidal stimulation improves parkinsonian motor signs and modulates neuronal activity throughout the basal ganglia thalamic network. Experimental neurology. 2012;233(1):581–586. doi: 10.1016/j.expneurol.2011.09.031 22001773 PMC3536483

[33] Lang AE, Zadikoff C. Parkinsonian tremor. Neurological disease and therapy. 2005;70:195.

[34] Gillies A, Willshaw D, Li Z. Subthalamic–pallidal interactions are critical in determining normal and abnormal functioning of the basal ganglia. Proceedings of the Royal Society of London Series B: Biological Sciences. 2002;269(1491):545–551. doi: 10.1098/rspb.2001.1817 11916469 PMC1690930

[35] Kumar A, Cardanobile S, Rotter S, Aertsen A. The role of inhibition in generating and controlling Parkinson’s disease oscillations in the basal ganglia. Frontiers in systems neuroscience. 2011;5:86. doi: 10.3389/fnsys.2011.00086 22028684 PMC3199726

[36] Pasillas-Lépine W. Delay-induced oscillations in Wilson and Cowan’s model: an analysis of the subthalamo-pallidal feedback loop in healthy and parkinsonian subjects. Biological cybernetics. 2013;107(3):289–308. doi: 10.1007/s00422-013-0549-3 23400597

[37] Merrison-Hort R, Borisyuk R. The emergence of two anti-phase oscillatory neural populations in a computational model of the Parkinsonian globus pallidus. Frontiers in computational neuroscience. 2013;7:173. doi: 10.3389/fncom.2013.00173 24348374 PMC3844854

[38] Holgado AJN, Terry JR, Bogacz R. Conditions for the Generation of Beta Oscillations in the Subthalamic Nucleus–Globus Pallidus Network. Journal of Neuroscience. 2010;30(37):12340–12352. doi: 10.1523/JNEUROSCI.0817-10.2010 20844130 PMC6633459

[39] Holt AB, Netoff TI. Origins and suppression of oscillations in a computational model of Parkinson’s disease. Journal of computational neuroscience. 2014;37(3):505–521. doi: 10.1007/s10827-014-0523-7 25099916 PMC4225169

[40] Leblois A, Boraud T, Meissner W, Bergman H, Hansel D. Competition between Feedback Loops Underlies Normal and Pathological Dynamics in the Basal Ganglia. Journal of Neuroscience. 2006;26(13):3567–3583. doi: 10.1523/JNEUROSCI.5050-05.2006 16571765 PMC6673853

[41] van Albada SJ, Robinson PA. Mean-field modeling of the basal ganglia-thalamocortical system. I: Firing rates in healthy and parkinsonian states. Journal of Theoretical Biology. 2009;257(4):642–663. doi: 10.1016/j.jtbi.2008.12.018 19168074

[42] Damodaran S, Cressman JR, Jedrzejewski-Szmek Z, Blackwell KT. Desynchronization of fast-spiking interneurons reduces *β*-band oscillations and imbalance in firing in the dopamine-depleted striatum. Journal of Neuroscience. 2015;35(3):1149–1159. doi: 10.1523/JNEUROSCI.3490-14.2015 25609629 PMC4300321

[43] van Albada SJ, Gray RT, Drysdale PM, Robinson PA. Mean-field modeling of the basal ganglia-thalamocortical system. II: dynamics of parkinsonian oscillations. Journal of theoretical biology. 2009;257(4):664–688. doi: 10.1016/j.jtbi.2008.12.013 19154745

[44] Zemel D, Gritton H, Cheung C, Shankar S, Kramer M, Han X. Dopamine depletion selectively disrupts interactions between striatal neuron subtypes and LFP oscillations. Cell reports. 2022;38(3):110265. doi: 10.1016/j.celrep.2021.110265 35045299 PMC8820590

[45] Mallet N, Micklem BR, Henny P, Brown MT, Williams C, Bolam JP, et al. Dichotomous Organization of the External Globus Pallidus. Neuron. 2012;74(6):1075–1086. doi: 10.1016/j.neuron.2012.04.027 22726837 PMC3407962

[46] Abdi A, Mallet N, Mohamed FY, Sharott A, Dodson PD, Nakamura KC, et al. Prototypic and Arkypallidal Neurons in the Dopamine-Intact External Globus Pallidus. Journal of Neuroscience. 2015;35(17):6667–6688. doi: 10.1523/JNEUROSCI.4662-14.2015 25926446 PMC4412890

[47] Fujiyama F, Nakano T, Matsuda W, Furuta T, Udagawa J, Kaneko T. A single-neuron tracing study of arkypallidal and prototypic neurons in healthy rats. Brain Structure and Function. 2016;221(9):4733–4740. doi: 10.1007/s00429-015-1152-2 26642797

[48] Bevan MD, Booth PA, Eaton SA, Bolam JP. Selective innervation of neostriatal interneurons by a subclass of neuron in the globus pallidus of the rat. Journal of Neuroscience. 1998;18(22):9438–9452. doi: 10.1523/JNEUROSCI.18-22-09438.1998 9801382 PMC6792890

[49] Crompe Bdl, Aristieta A, Leblois A, Elsherbiny S, Boraud T, Mallet NP. The globus pallidus orchestrates abnormal network dynamics in a model of Parkinsonism. Nature Communications. 2020;11(1):1570. doi: 10.1038/s41467-020-15352-3 32218441 PMC7099038

[50] Dodson PD, Larvin JT, Duffell JM, Garas FN, Doig NM, Kessaris N, et al. Distinct Developmental Origins Manifest in the Specialized Encoding of Movement by Adult Neurons of the External Globus Pallidus. Neuron. 2015;86(2):501–513. doi: 10.1016/j.neuron.2015.03.007 25843402 PMC4416107

[51] Hernández VM, Hegeman DJ, Cui Q, Kelver DA, Fiske MP, Glajch KE, et al. Parvalbumin+ Neurons and Npas1+ Neurons Are Distinct Neuron Classes in the Mouse External Globus Pallidus. Journal of Neuroscience. 2015;35(34):11830–11847. doi: 10.1523/JNEUROSCI.4672-14.2015 26311767 PMC4549397

[52] Mastro KJ, Bouchard RS, Holt HA, Gittis AH. Transgenic mouse lines subdivide external segment of the globus pallidus (GPe) neurons and reveal distinct GPe output pathways. Journal of Neuroscience. 2014;34(6):2087–2099. doi: 10.1523/JNEUROSCI.4646-13.2014 24501350 PMC3913864

[53] Cagnan H, Mallet N, Moll CKE, Gulberti A, Holt AB, Westphal M, et al. Temporal evolution of beta bursts in the parkinsonian cortical and basal ganglia network. Proceedings of the National Academy of Sciences. 2019;116(32):16095–16104. doi: 10.1073/pnas.1819975116 31341079 PMC6690030

[54] Wilson CJ. What controls the timing of striatal spiny cell action potentials in the up state? In: The Basal Ganglia IX. Springer; 2009. p. 49–61.

[55] Gage GJ, Stoetzner CR, Wiltschko AB, Berke JD. Selective activation of striatal fast-spiking interneurons during choice execution. Neuron. 2010;67(3):466–479. doi: 10.1016/j.neuron.2010.06.034 20696383 PMC2920892

[56] Dong J, Hawes S, Wu J, Le W, Cai H. Connectivity and Functionality of the Globus Pallidus Externa Under Normal Conditions and Parkinson’s Disease. Frontiers in Neural Circuits. 2021;15. doi: 10.3389/fncir.2021.645287 33737869 PMC7960779

[57] Corbit VL, Whalen TC, Zitelli KT, Crilly SY, Rubin JE, Gittis AH. Pallidostriatal Projections Promote beta Oscillations in a Dopamine-Depleted Biophysical Network Model. Journal of Neuroscience. 2016;36(20):5556–5571. doi: 10.1523/JNEUROSCI.0339-16.2016 27194335 PMC4871989

[58] Lindahl M, Kotaleski JH. Untangling basal ganglia network dynamics and function: Role of dopamine depletion and inhibition investigated in a spiking network model. Eneuro. 2016;3(6). doi: 10.1523/ENEURO.0156-16.2016 28101525 PMC5228592

[59] Oorschot DE. Total number of neurons in the neostriatal, pallidal, subthalamic, and substantia nigral nuclei of the rat basal ganglia: a stereological study using the cavalieri and optical disector methods. Journal of Comparative Neurology. 1996;366(4):580–599. doi: 10.1002/(SICI)1096-9861(19960318)366:4<580::AID-CNE3>3.0.CO;2-0 8833111

[60] Saunders A, Huang KW, Sabatini BL. Globus pallidus externus neurons expressing parvalbumin interconnect the subthalamic nucleus and striatal interneurons. PloS one. 2016;11(2):e0149798. doi: 10.1371/journal.pone.0149798 26905595 PMC4764347

[61] Steiner H, Tseng KY. Handbook of basal ganglia structure and function. Academic Press; 2016.

[62] Berke JD, Okatan M, Skurski J, Eichenbaum HB. Oscillatory entrainment of striatal neurons in freely moving rats. Neuron. 2004;43(6):883–896. doi: 10.1016/j.neuron.2004.08.035 15363398

[63] Miller BR, Walker AG, Shah AS, Barton SJ, Rebec GV. Dysregulated information processing by medium spiny neurons in striatum of freely behaving mouse models of Huntington’s disease. Journal of neurophysiology. 2008;100(4):2205–2216. doi: 10.1152/jn.90606.2008 18667541 PMC2576201

[64] Fourcaud N, Brunel N. Dynamics of the firing probability of noisy integrate-and-fire neurons. Neural computation. 2002;14(9):2057–2110. doi: 10.1162/089976602320264015 12184844

[65] Burkitt AN. A review of the integrate-and-fire neuron model: II. Inhomogeneous synaptic input and network properties. Biological cybernetics. 2006;95(2):97–112. doi: 10.1007/s00422-006-0082-8 16821035

[66] Meffin H, Burkitt AN, Grayden DB. An analytical model for the ‘large, fluctuating synaptic conductance state’typical of neocortical neurons in vivo. Journal of computational neuroscience. 2004;16(2):159–175. doi: 10.1023/B:JCNS.0000014108.03012.81 14758064

[67] Cavallari S, Panzeri S, Mazzoni A. Comparison of the dynamics of neural interactions between current-based and conductance-based integrate-and-fire recurrent networks. Frontiers in neural circuits. 2014;8:12. doi: 10.3389/fncir.2014.00012 24634645 PMC3943173

[68] Fourcaud-Trocmé N, Hansel D, Van Vreeswijk C, Brunel N. How spike generation mechanisms determine the neuronal response to fluctuating inputs. Journal of neuroscience. 2003;23(37):11628–11640. doi: 10.1523/JNEUROSCI.23-37-11628.2003 14684865 PMC6740955

[69] Ermentrout GB, Kopell N. Parabolic bursting in an excitable system coupled with a slow oscillation. SIAM journal on applied mathematics. 1986;46(2):233–253. doi: 10.1137/0146017

[70] Izhikevich EM. Simple model of spiking neurons. IEEE Transactions on neural networks. 2003;14(6):1569–1572. doi: 10.1109/TNN.2003.820440 18244602

[71] Brette R, Gerstner W. Adaptive exponential integrate-and-fire model as an effective description of neuronal activity. Journal of neurophysiology. 2005;94(5):3637–3642. doi: 10.1152/jn.00686.2005 16014787

[72] Baufreton J, Kirkham E, Atherton JF, Menard A, Magill PJ, Bolam JP, et al. Sparse but selective and potent synaptic transmission from the globus pallidus to the subthalamic nucleus. Journal of neurophysiology. 2009;. doi: 10.1152/jn.00305.2009 19458148 PMC2712268

[73] Brunel N, Wang XJ. What determines the frequency of fast network oscillations with irregular neural discharges? I. Synaptic dynamics and excitation-inhibition balance. Journal of neurophysiology. 2003;90(1):415–430. doi: 10.1152/jn.01095.2002 12611969

[74] Fan KY, Baufreton J, Surmeier DJ, Chan CS, Bevan MD. Proliferation of external globus pallidus-subthalamic nucleus synapses following degeneration of midbrain dopamine neurons. Journal of Neuroscience. 2012;32(40):13718–13728. doi: 10.1523/JNEUROSCI.5750-11.2012 23035084 PMC3475197

[75] Gittis AH, Hang GB, LaDow ES, Shoenfeld LR, Atallah BV, Finkbeiner S, et al. Rapid target-specific remodeling of fast-spiking inhibitory circuits after loss of dopamine. Neuron. 2011;71(5):858–868. doi: 10.1016/j.neuron.2011.06.035 21903079 PMC3170520

[76] Blesa J, Trigo-Damas I, Dileone M, Del Rey NLG, Hernandez LF, Obeso JA. Compensatory mechanisms in Parkinson’s disease: circuits adaptations and role in disease modification. Experimental neurology. 2017;298:148–161. doi: 10.1016/j.expneurol.2017.10.002 28987461

[77] Yelnik J, Percheron G. Subthalamic neurons in primates: a quantitative and comparative analysis. Neuroscience. 1979;4(11):1717–1743. doi: 10.1016/0306-4522(79)90030-7 117397

[78] Shepherd GM. Corticostriatal connectivity and its role in disease. Nature Reviews Neuroscience. 2013;14(4):278–291. doi: 10.1038/nrn3469 23511908 PMC4096337

[79] Verstynen TD, Badre D, Jarbo K, Schneider W. Microstructural organizational patterns in the human corticostriatal system. Journal of neurophysiology. 2012;107(11):2984–2995. doi: 10.1152/jn.00995.2011 22378170 PMC4073961

[80] Hardman CD, Henderson JM, Finkelstein DI, Horne MK, Paxinos G, Halliday GM. Comparison of the basal ganglia in rats, marmosets, macaques, baboons, and humans: volume and neuronal number for the output, internal relay, and striatal modulating nuclei. Journal of Comparative Neurology. 2002;445(3):238–255. doi: 10.1002/cne.10165 11920704

[81] Butcher J. Practical Runge–Kutta methods for scientific computation. The ANZIAM Journal. 2009;50(3):333–342. doi: 10.1017/S1446181109000030

[82] O’Neill ME. PCG: A family of simple fast space-efficient statistically good algorithms for random number generation. ACM Transactions on Mathematical Software. 2014.

[83] Terman D, Rubin JE, Yew A, Wilson C. Activity patterns in a model for the subthalamopallidal network of the basal ganglia. Journal of Neuroscience. 2002;22(7):2963–2976. doi: 10.1523/JNEUROSCI.22-07-02963.2002 11923461 PMC6758326

[84] Suryanarayana SM, Hellgren Kotaleski J, Grillner S, Gurney KN. Roles for globus pallidus externa revealed in a computational model of action selection in the basal ganglia. Neural Networks. 2019;109:113–136. doi: 10.1016/j.neunet.2018.10.003 30414556

[85] Blenkinsop A, Anderson S, Gurney K. Frequency and function in the basal ganglia: the origins of beta and gamma band activity. The Journal of Physiology. 2017;595(13):4525–4548. doi: 10.1113/JP273760 28334424 PMC5491879

[86] Chakravarty K, Roy S, Sinha A, Nambu A, Chiken S, Hellgren Kotaleski J, et al. Transient Response of Basal Ganglia Network in Healthy and Low-Dopamine State. eNeuro. 2022;9(2):ENEURO.0376–21.2022. doi: 10.1523/ENEURO.0376-21.2022 35140075 PMC8938981

[87] Pavlides A, Hogan SJ, Bogacz R. Computational models describing possible mechanisms for generation of excessive beta oscillations in Parkinson’s disease. PLoS computational biology. 2015;11(12):e1004609. doi: 10.1371/journal.pcbi.1004609 26683341 PMC4684204

[88] Azizpour Lindi S, Mallet NP, Leblois A. Synaptic changes in pallidostriatal circuits observed in parkinsonian model triggers abnormal beta synchrony with accurate spatio-temporal properties across the basal ganglia. bioRxiv. 2023; p. 2023–03. 10.1523/JNEUROSCI.0419-23.2023 PMC10903930 38123981

[89] Adam EM, Brown EN, Kopell N, McCarthy MM. Deep brain stimulation in the subthalamic nucleus for Parkinson’s disease can restore dynamics of striatal networks. Proceedings of the National Academy of Sciences. 2022;119(19):e2120808119. doi: 10.1073/pnas.2120808119 35500112 PMC9171607

[90] Oswal A, Cao C, Yeh CH, Neumann WJ, Gratwicke J, Akram H, et al. Neural signatures of hyperdirect pathway activity in Parkinson’s disease. Nature communications. 2021;12(1):5185. doi: 10.1038/s41467-021-25366-0 34465771 PMC8408177

[91] Iskhakova L, Rappel P, Deffains M, Fonar G, Marmor O, Paz R, et al. Modulation of dopamine tone induces frequency shifts in cortico-basal ganglia beta oscillations. Nature communications. 2021;12(1):7026. doi: 10.1038/s41467-021-27375-5 34857767 PMC8640051

[92] Sharott A, Magill PJ, Harnack D, Kupsch A, Meissner W, Brown P. Dopamine depletion increases the power and coherence of *β*-oscillations in the cerebral cortex and subthalamic nucleus of the awake rat. European Journal of Neuroscience. 2005;21(5):1413–1422. doi: 10.1111/j.1460-9568.2005.03973.x 15813951

[93] Rubin JE. Computational models of basal ganglia dysfunction: the dynamics is in the details. Current opinion in neurobiology. 2017;46:127–135. doi: 10.1016/j.conb.2017.08.011 28888856

[94] Moran RJ, Mallet N, Litvak V, Dolan RJ, Magill PJ, Friston KJ, et al. Alterations in brain connectivity underlying beta oscillations in Parkinsonism. PLoS computational biology. 2011;7(8):e1002124. doi: 10.1371/journal.pcbi.1002124 21852943 PMC3154892

[95] Lai HY, Liao LD, Lin CT, Hsu JH, He X, Chen YY, et al. Design, simulation and experimental validation of a novel flexible neural probe for deep brain stimulation and multichannel recording. Journal of neural engineering. 2012;9(3):036001. doi: 10.1088/1741-2560/9/3/036001 22488106

[96] Butenko K, Bahls C, Schröder M, Köhling R, van Rienen U. OSS-DBS: Open-source simulation platform for deep brain stimulation with a comprehensive automated modeling. PLoS computational biology. 2020;16(7):e1008023. doi: 10.1371/journal.pcbi.1008023 32628719 PMC7384674

[97] Fleming JE, Dunn E, Lowery MM. Simulation of closed-loop deep brain stimulation control schemes for suppression of pathological beta oscillations in Parkinson’s disease. Frontiers in neuroscience. 2020;14:166. doi: 10.3389/fnins.2020.00166 32194372 PMC7066305

[98] West TO, Magill PJ, Sharott A, Litvak V, Farmer SF, Cagnan H. Stimulating at the right time to recover network states in a model of the cortico-basal ganglia-thalamic circuit. PLoS computational biology. 2022;18(3):e1009887. doi: 10.1371/journal.pcbi.1009887 35245281 PMC8939795

[99] Tinkhauser G. The present and future role of clinical neurophysiology for Deep Brain Stimulation; 2022. 10.1016/j.clinph.2022.05.012 35717329

[100] Shah A, Nguyen TAK, Peterman K, Khawaldeh S, Debove I, Shah SA, et al. Combining multimodal biomarkers to guide deep brain stimulation programming in Parkinson disease. Neuromodulation: technology at the neural interface. 2022;. doi: 10.1016/j.neurom.2022.01.017 35219571 PMC7614142

[101] Tinkhauser G, Moraud EM. Controlling Clinical States Governed by Different Temporal Dynamics With Closed-Loop Deep Brain Stimulation: A Principled Framework. Frontiers in neuroscience. 2021;15. doi: 10.3389/fnins.2021.734186 34858126 PMC8632004

[102] Crowell AL, Ryapolova-Webb ES, Ostrem JL, Galifianakis NB, Shimamoto S, Lim DA, et al. Oscillations in sensorimotor cortex in movement disorders: an electrocorticography study. Brain. 2012;135(2):615–630. doi: 10.1093/brain/awr332 22252995 PMC3281473

[103] Vissani M, Palmisano C, Volkmann J, Pezzoli G, Micera S, Isaias IU, et al. Impaired reach-to-grasp kinematics in parkinsonian patients relates to dopamine-dependent, subthalamic beta bursts. npj Parkinson’s Disease. 2021;7(1):53. doi: 10.1038/s41531-021-00187-6 34188058 PMC8242004

[104] Canessa A, Palmisano C, Isaias IU, Mazzoni A. Gait-related frequency modulation of beta oscillatory activity in the subthalamic nucleus of parkinsonian patients. Brain Stimulation. 2020;13(6):1743–1752. doi: 10.1016/j.brs.2020.09.006 32961337

[105] López-Azcárate J, Tainta M, Rodríguez-Oroz MC, Valencia M, González R, Guridi J, et al. Coupling between beta and high-frequency activity in the human subthalamic nucleus may be a pathophysiological mechanism in Parkinson’s disease. Journal of Neuroscience. 2010;30(19):6667–6677. doi: 10.1523/JNEUROSCI.5459-09.2010 20463229 PMC6632566

[106] Oswal A, Beudel M, Zrinzo L, Limousin P, Hariz M, Foltynie T, et al. Deep brain stimulation modulates synchrony within spatially and spectrally distinct resting state networks in Parkinson’s disease. Brain. 2016;139(5):1482–1496. doi: 10.1093/brain/aww048 27017189 PMC4845255

