# Supplementary material for: Dopamine depletion leads to pathological synchronization of distinct basal ganglia loops in the beta band
Source: PLoS Comput Biol. 2023 Apr 27;19(4):e1010645. doi: 10.1371/journal.pcbi.1010645 (PMC10168586; doi:10.1371/journal.pcbi.1010645)
Supplement: S1 Text — This file collects the following supporting tables and figures: Table A. Parameter changes from the Complete to the Simplified Model. Fig A. Effects of the coupling strength ε on the Simplified Model: plots for the D2 nucleus. Fig B. Effects of Dopamine Depletion Dd on the Simplified Model (ε = 0.75): plots for the STN nucleus. Fig C. Effects of Dopamine Depletion Dd on the Complete Model: plot for the STN nucleus. Fig D. Effects of the connectivity alterations due to Dopamine Depletion on the intensity of β activity. Fig E. Robustness of the results to alterations of the connectivity parameters and neuron and population parameters. Fig F. Probability distribution of the instantaneous intensity of STN activity for different degrees of Dopamine Depletion. (PDF) [file pcbi.1010645.s001.pdf]

S1 Text - Supporting Information

Contents

1 Parameter changes from the Complete to the Simplified Model 1

2 D2 nucleus PSD as function of ε interaction parameter 2

3 STN nucleus PSD as function of D_d parameter in the Simplified Model 3

4 STN nucleus PSD as function of D_d parameter in the Complete Model 4

5 Effects on pathological oscillations of connectivity alterations due to dopamine depletion 5

6 Robustness of pathological oscillations onset to alterations of model parameters 7

7 Probability distribution of the instantaneous intensity of the STN activity for different degrees of Dopamine Depletion 9

1 Parameter changes from the Complete to the Simplified Model

Population	Parameter	Adapted-value
FSN	syn. weight from GPe-TI,GPe-TA and GPTI*	0.85 nS (inhib.)
	ν_{ext}	0.787 kHz
	I_e	-57 pA
D2	syn. weight from FSN	2.55 nS (inhib.)
	I_e	-92 pA
GPTI-A	syn. weight from D2	0.68 nS (inhib.)
	I_e	-160 pA
GPTI-B	syn. weight from D2	0.68 nS (inhib.)
	I_e	-160 pA
D2*	I_e	-136 pA
GPTI*	I_e	-112 pA
STN*	I_e	-20 pA

Table A: Parameter changes from the Complete to the Simplified Model.

2 D2 nucleus PSD as function of ε interaction parameter

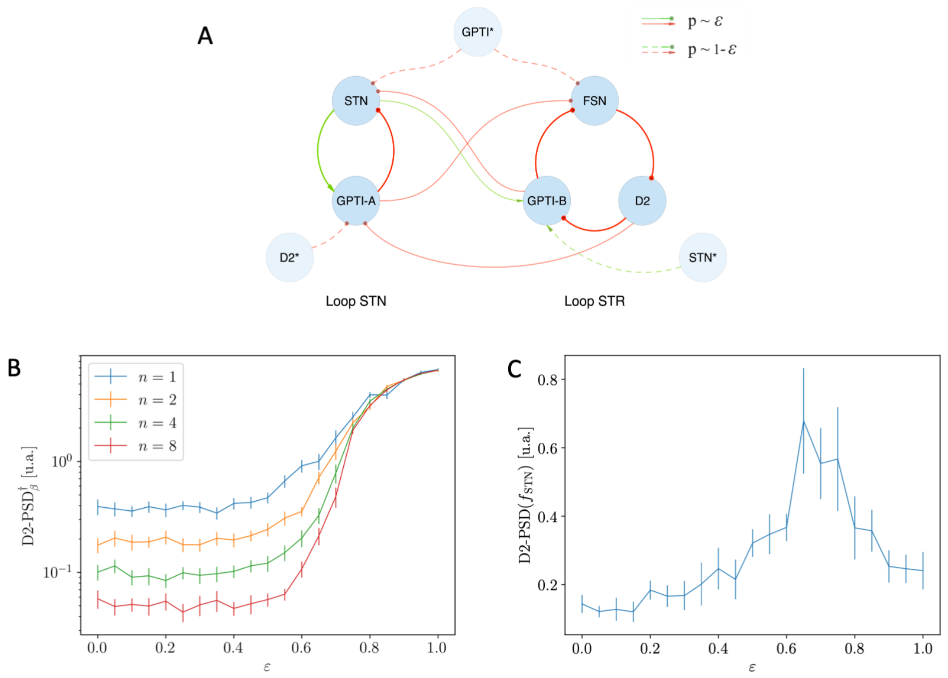

Fig A: **Effects of the coupling strength ε on the Simplified Model: plots for the D2 nucleus (A)** Schematic representation of the Simplified model: the modulation of the coupling strength between the STR and STN loops is obtained by varying the connection probabilities $p(\varepsilon, S \to T) = \varepsilon p_{1,S\to T}$ (see Eq. (2)) of *inter*-loops connections. **(B)** Unbiased measure of the intensity of D2 β activity as a function of ε for different values of the population size n. **(C)** Value of the D2-PSD nuclei computed at the natural frequency of the STN oscillator as a function of the coupling strength. These results are associated with the analogous in panels D and E of Fig 6.

3 STN nucleus PSD as function of D_d parameter in the Simplified Model

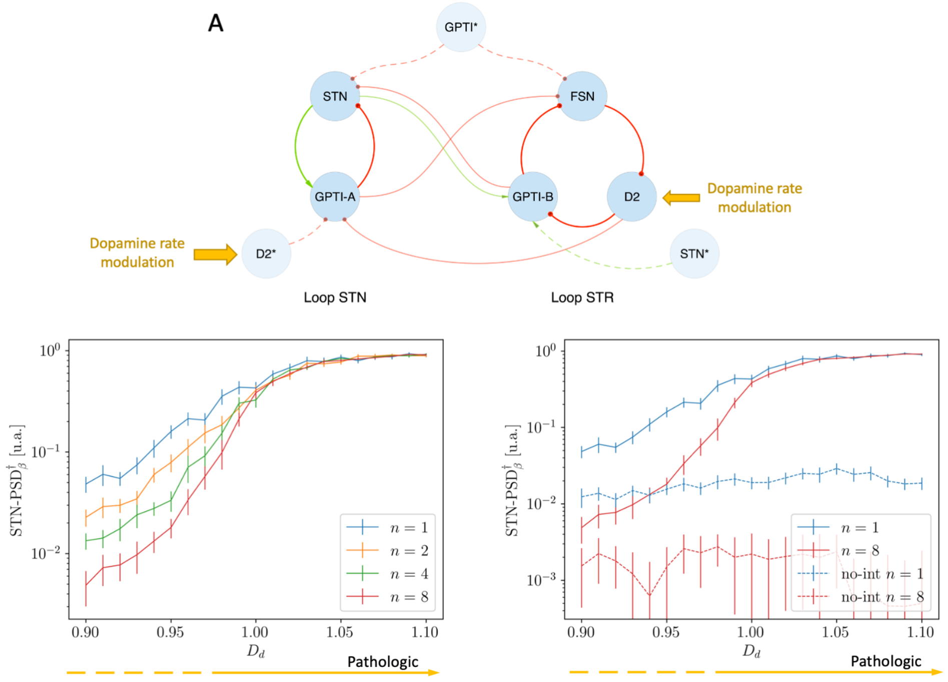

Fig B: **Effects of Dopamine Depletion D_d on the Simplified Model ($\varepsilon = 0.75$): plots for the STN nucleus.** **(A)** Schematic representation of the Simplified model in the interacting case: the targets of Dopamine modulation are highlighted. **(B)** Unbiased measure of the intensity of the STN β activity as a function of D_d for different values of the population size n. **(C)** Unbiased measure of the intensity of the STN β activity as a function of D_d for $n = 1$ (blue) and $n = 8$ (red) and comparison between the interacting ($\varepsilon = 0.75$, continuous lines) and non-interacting condition ($\varepsilon = 0.00$, dashed lines): the intensity of β activity is preserved if and only if the two oscillators are synchronized. Compare these results with the analogous in panels D and E of Fig 8.

4 STN nucleus PSD as function of D_d parameter in the Complete Model

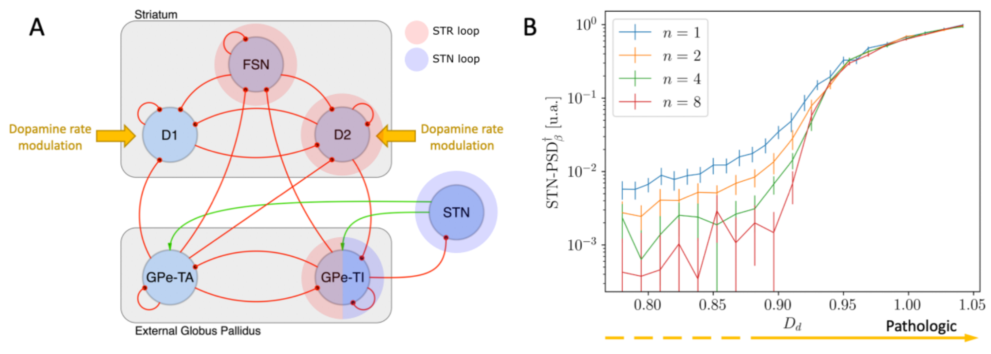

Fig C: **Effects of Dopamine Depletion D_d on the Complete Model: plot for the STN nucleus.** (A) Schematic representation of the Complete Model: the targets of Dopamine modulation are highlighted. **(B)** Unbiased measure of the intensity of the STN β activity as a function of D_d for different values of the population size n. Compare these results with the analogous in panel D of Fig 9.

5 Effects on pathological oscillations of connectivity alterations due to dopamine depletion

Throughout this work, the condition of Dopamine Depletion is modeled as the complementary modulation of the mean discharge rate of the D1 and D2 populations in the Striatum (see Figure 2 in the main text).
However, several works [57, 74-76] showed that dopamine depletion leads also to alterations in Basal Ganglia connectivity. In mice, as a result of dopamine depletion, the number of GPe–STN synapses doubles with no alteration in synapse size and efficacy [74] and FSN cells double their connectivity to D2 MSNs, whereas connections to D1 MSNs remain unchanged [75]. In this section we investigate the effects of a more detailed modelization of dopamine depletion on STR and STN loop, accounting also for these evidences. In addition to the modulation of the external input rate to the striatal populations (see equation 3), dopamine depletion is here modeled by modulating the connection probabilities $p_{\mathrm{FSN}\to\mathrm{D2}}$ and $p_{\text{GPe-TI}\to\mathrm{STN}}$ (see panel A in Figure D) according to:

$$p_{\mathrm{FSN}\to\mathrm{D2}}(D^*_d) = p_{\mathrm{FSN}\to\mathrm{D2},1}\left[1 + \frac{1}{D^*_{d,max} - D^*_{d,min}}(D^*_d - D^*_{d,min})\right]$$

$$p_{\text{GPe-TI}\to\mathrm{STN}}(D^*_d) = p_{\text{GPe-TI}\to\mathrm{STN},1}\left[1 + \frac{1}{D^*_{d,max} - D^*_{d,min}}(D^*_d - D^*_{d,min})\right]$$

where $p_{\mathrm{FSN}\to\mathrm{D2},1}$ and $p_{\text{GPe-TI}\to\mathrm{STN},1}$ are the reference values indicated in Table 3, $D^*_{d,min} = 0.9$ and $D^*_{d,min} = 1.1$.

Taking into account also dopamine depletion effects on connectivity, the intensity of β activity increases in the STR loop (panel B in Figure D), in a very similar way to what observed taking into account only the effects dopmamine depletion effects on D1 and D2 rate (panel C in Figure D). The increase in β activity observed in the STN loop is smaller than what observed in the STR loop (panel D in Figure D), but still different from what is observed when modeling only dopamine depletion effects on rate, in which no modulation was observed (panel E in Figure D). However the intensity of β activity is not robust in the limit of large n. This suggests that the introduction of a more detailed modelization of dopamine depletion, including also the strenghtening of the GPE $\to$ STN and FSN$\to$D2 connectivity, is not sufficient to account for the emergence of prominent β activity within the isolated oscillators. As shown by the results in the main text, the key element for the emergence of prominent β activity is not determined by these alterations, but lies in the interplay between the two identified oscillators. For the sake of simplicity in results interpretations, we did not include then dopamine-dependent connectivity alterations in the main text as they would not change the presented results on a qualitative description, but only affect minor details at a quantitative level.

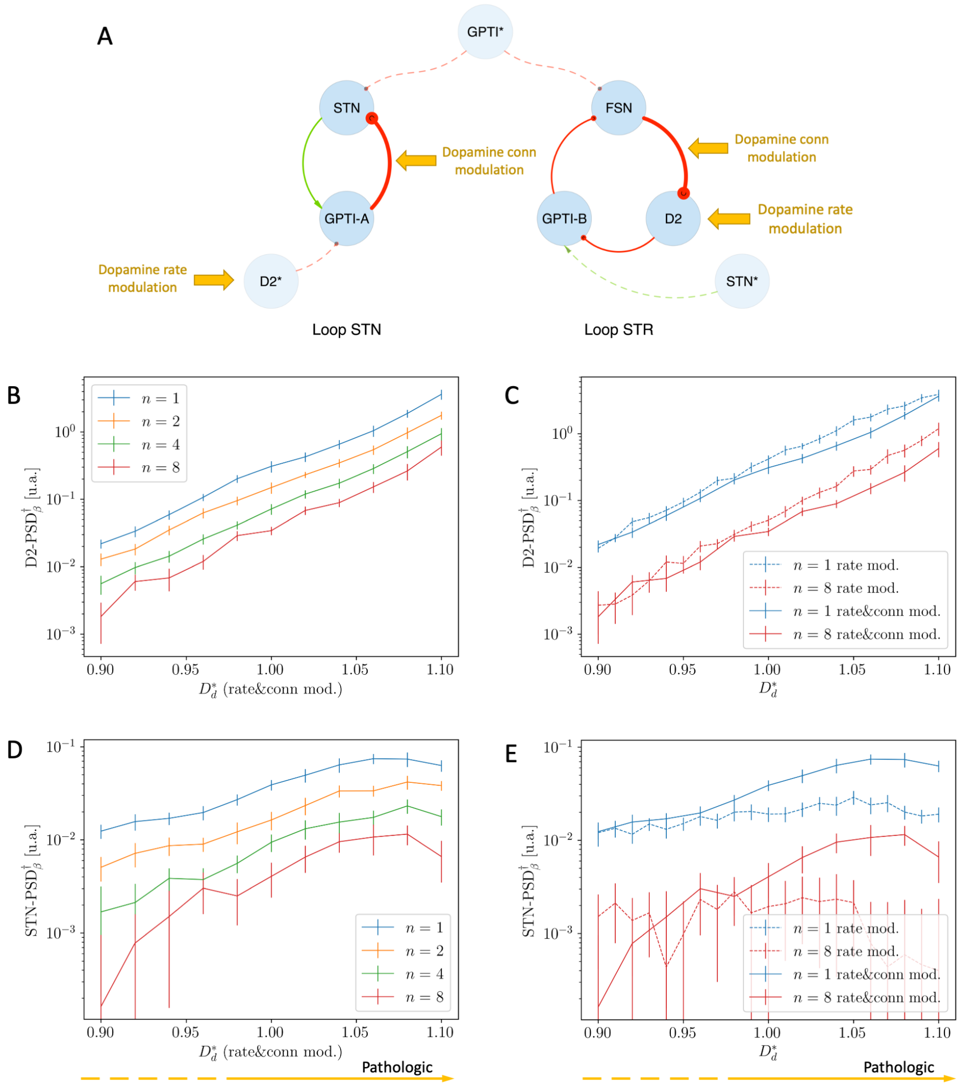

Fig D: **Effects of the connectivity alterations due to Dopamine Depletion on the intensity of β activity.** **(A)** Schematic representation of the Simplified model in the interacting case with the modelization of Dopamine Depletion including connectivity alterations. **(B)** Unbiased measure of the intensity of β oscillations as a function of D_d^* for different values of n and for the D2 population. **(C)** Comparison between the intensity of β oscillations when D_d^* alters only the rate of D1 and D2 (dashed lines) or both the rate and the connectivity (continous lines), for $n \in [1, 8]$. **(D)** same as (B) for STN. **(E)** Same as (C) for STN.

6 Robustness of pathological oscillations onset to alterations of model parameters

In order to test whether the main result of our work, i.e., the process of synchronization associated with dopamine depletion, critically depends on the choice of the model parameters, we repeated the analysis after small alterations of the model parameters. We first consider the connectivity properties of the model and slightly alter the value of the parameters regulating the connection probability p_i and the synaptic weight w_i of each connection i in the model. Particularly, for each connection i, we considered the pair (p_i, w_i) and, in order to maintain the mean firing activity of the different nuclei in the realistic range, we jittered these parameters according to:

$$w_i' = w_i \cdot x_i \qquad\qquad p_i' = p_i/x_i$$

with $\{x_i\}_i$ independently extracted for each connection from a gaussian distribution with mean $\mu = 1$ and a standard deviation $\sigma = 0.1$.
We repeated the extraction of the $\{x_i\}_i$ values four times independently and, for each of these extractions we performed the analysis on the intensity of β activity as a function of D_d. The results of this study are reported in panels A and B of Figure E, and show that, despite small variations, the process of synchronization and the emergence of prominent β activity is robust to the described alterations.

We now consider the network parameters regulating the neuron model and the size of each population k in our model. Particularly, in order to maintain the mean firing activity of the different nuclei in the realistic range, we jittered the parameters:

- N_k, $\tau_{ex,k}$, $\tau_{in,k}$, $\nu_{ext,k}$ according to:

  $$N_k' = N_k \cdot x \qquad \tau_{ex,k}' = \tau/x$$
  $$\nu_{ext,k}' = \nu_{ext,k} \cdot x \qquad \tau_{in,k}' = \tau/x$$

  with x extracted from a gaussian distribution with mean $\mu = 1$ and a standard deviation $\sigma = 0.1$

- `dev_ext_weight`$_k$, $V_{res,k}$, $t_{ref,k}$, $V_{peak,k}$, $E_{L,k}$, $E_{ex,k}$, $E_{in,k}$, $I_{e,k}$, $C_{m,k}$, $g_{L,k}$, and the adaptation parameters a_k, b_k and $\tau_{w,k}$ according to:

  $$E_{L,k}' = E_{L,k} \cdot y_{0,k} \qquad \texttt{dev\_ext\_weight}_k{}' = \texttt{dev\_ext\_weight}_k \cdot x_{0,k}$$
  $$E_{ex,k}' = E_{ex,k} \cdot y_{1,k} \qquad V_{res,k}' = V_{res,k} \cdot x_{1,k}$$
  $$E_{in,k}' = E_{in,k} \cdot y_{2,k} \qquad V_{res,k}' = V_{res,k} \cdot x_{2,k}$$
  $$I_{e,k}' = I_{e,k} \cdot y_{3,k}$$
  $$C_{m,k}' = C_{m,k} \cdot y_{4,k}$$
  $$g_{L,k}' = g_{L,k} \cdot y_{5,k}$$

  with $\{x_{j,k}\}_{j,k}$ and $\{y_{j,k}\}_{j,k}$ independently extracted for each population k from a gaussian distribution with mean $\mu = 1$ and a standard deviation $\sigma_x = 0.1$ and $\sigma_y = 0.01$ respectively.

In this case also, we repeated the extraction of the x, $\{x_{j,k}\}_{j,k}$ and $\{y_{j,k}\}_{j,k}$ four times and, for each of these extractions, we performed the analysis on the intensity of β activity as a function of D_d. Similarly to the previous case, the results of the study highlight that the process of synchronization and the emergence of prominent β activity is qualitatively robust to the considered alterations (see panels C and D in Figure E).

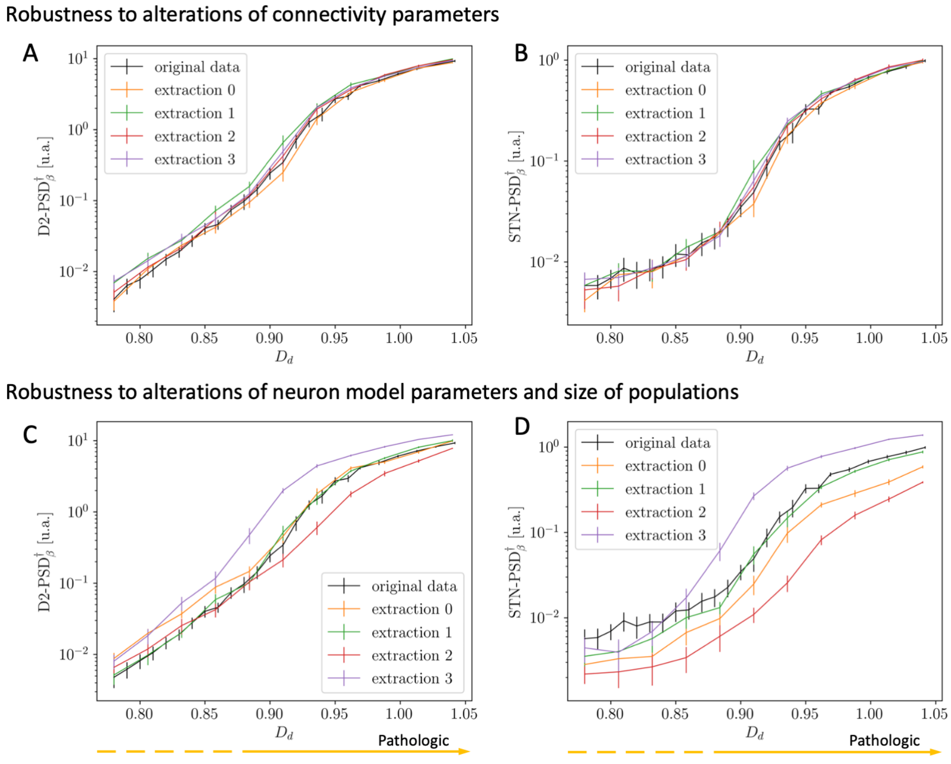

Fig E: **Robustness of the results to alterations of the connectivity parameters (A, B) and neuron and population parameters (C, D).** In all plots the unbiased measure of the intensity of β activity is reported for the original choice of parameters (see black lines and Tables 2 and 3 in the main text) and four independently jittered configurations of the parameters.

7 Probability distribution of the instantaneous intensity of the STN activity for different degrees of Dopamine Depletion

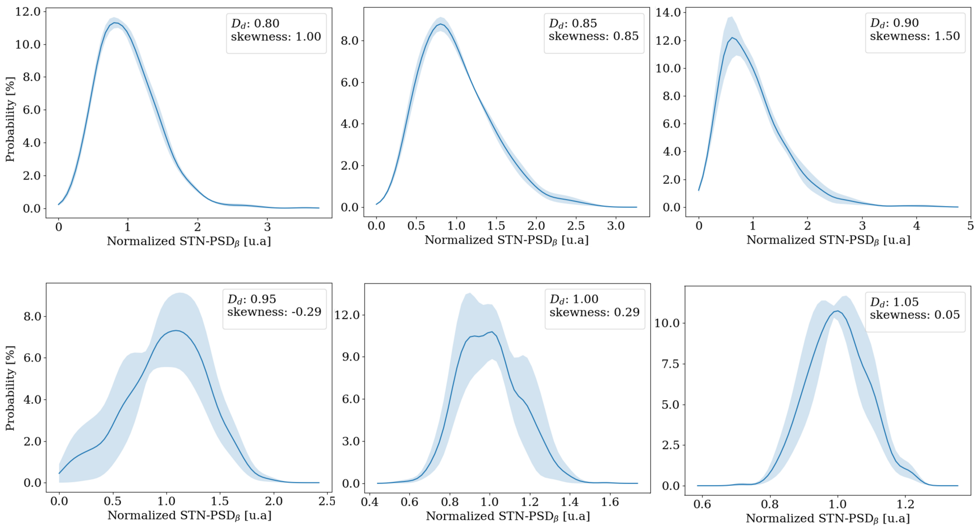

Fig F: **Probability distribution of the instantaneous intensity of STN activity for different degrees of Dopamine Depletion.** For each D_d, the x-axis represents the STN-PSD$_\beta$ normalized by its mean value. Solid lines and shaded areas represent the mean probability and the standard error of the mean, respectively.
